# Generation of a Nonbilayer Lipid Nanoenvironment after Epitope Binding Potentiates Neutralizing HIV-1 MPER Antibody

**DOI:** 10.1021/acsami.4c13353

**Published:** 2024-10-24

**Authors:** Sara Insausti, Ander Ramos-Caballero, Brian Wiley, Saul González-Resines, Johana Torralba, Anne Elizaga-Lara, Christine Shamblin, Akio Ojida, Jose M. M. Caaveiro, Michael B. Zwick, Edurne Rujas, Carmen Domene, José L. Nieva

**Affiliations:** † Instituto Biofisika (CSIC, UPV/EHU), University of the Basque Country (UPV/EHU), P.O. Box 644, Bilbao 48080, Spain; ‡ Department of Biochemistry and Molecular Biology, University of the Basque Country (UPV/EHU), P.O. Box 644, Bilbao 48080, Spain; § Department of Chemistry, University of Bath, Claverton Down, Bath BA2 7AX, United Kingdom; ⊥ Department of Immunology and Microbiology, The Scripps Research Institute, La Jolla, California 92037, United States; ¶ Department of Chemical Biology, School of Pharmaceutical Sciences, Kyushu University, Fukuoka 819-0395, Japan; □ Laboratory of Protein Drug Discovery, School of Pharmaceutical Sciences, Kyushu University, Fukuoka 819-0395, Japan; ■ Department of Pharmacy and Food Sciences, Faculty of Pharmacy, University of the Basque Country (UPV/EHU), Vitoria 01006, Spain; ○ Basque Foundation for Science, Ikerbasque, Bilbao48013, Spain

**Keywords:** antibody-membrane interaction, lipid nanoenvironment, membrane deformation, site-selective chemical modification, antibody engineering, HIV-1 antibody, molecular dynamics simulations, metadynamics

## Abstract

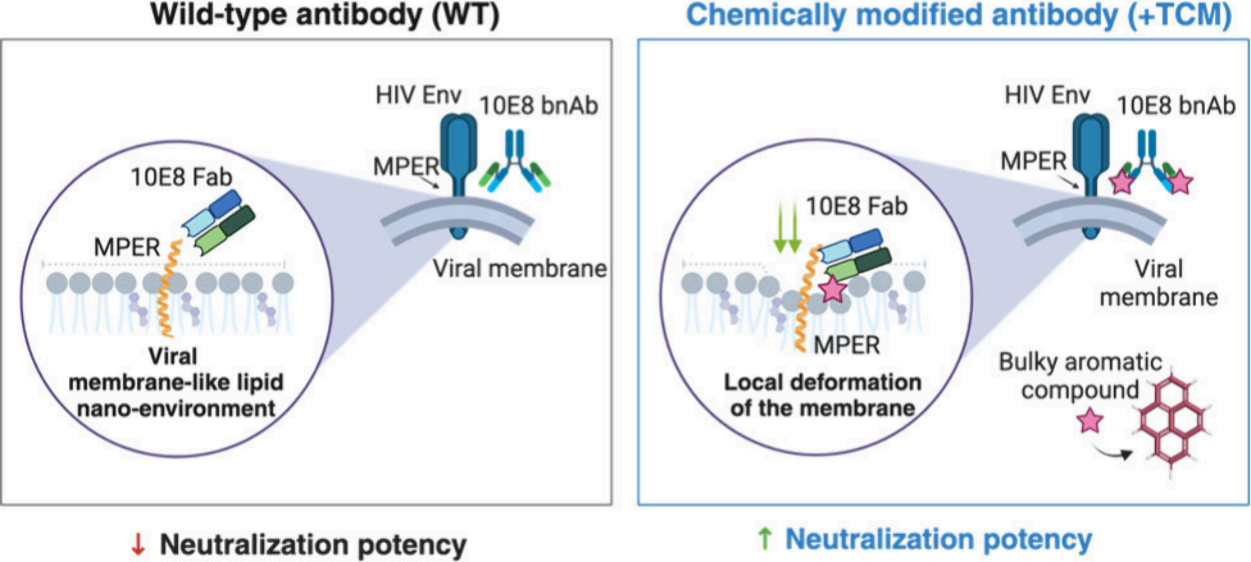

Establishment of interactions with the envelope lipids is a cardinal feature of broadly neutralizing antibodies (bnAbs) that recognize the Env membrane-proximal external region (MPER) of HIV. The lipid envelope constitutes a relevant component of the full “quinary” MPER epitope, and thus antibodies may be optimized through engineering their capacity to interact with lipids. However, the role of the chemically complex lipid nanoenvironment in the mechanism of MPER molecular recognition and viral neutralization remains poorly understood. To approach this issue, we computationally and experimentally investigated lipid interactions of broadly neutralizing antibody 10E8 and optimized versions engineered to enhance their epitope and membrane affinity by grafting bulky aromatic compounds. Our data revealed a correlation between neutralization potency and the establishment of favorable interactions with small headgroup lipids cholesterol and phosphatidylethanolamine, evolving after specific engagement with MPER. Molecular dynamics simulations of chemically modified Fabs in complex with an MPER-Transmembrane Domain helix supported the generation of a nanoenvironment causing localized deformation of the thick, rigid viral membrane and identified sphingomyelin preferentially occupying a phospholipid-binding site of 10E8. Together, these interactions appear to facilitate insertion of the Fabs through their engagement with the MPER epitope. These findings implicate individual lipid molecules in the neutralization function of MPER bnAbs, validate targeted chemical modification as a method to optimize MPER antibodies, and suggest pathways for MPER peptide-liposome vaccine development.

## 1 Introduction

Neutralizing antibodies elicited upon human immunodeficiency virus (HIV-1) infection drive the selection of virus escape mutants, launching a coevolutionary process involving HIV-1 variants and the host immune system. This process leads to extensive viral diversity within an individual and, occasionally, to the generation of broadly neutralizing antibodies (bnAbs).^1^ Attaining a high degree of potency and breadth by bnAbs requires prolonged exposure to viral antigens and is only achieved in 1% of individuals after years of persistent infection. In the absence of a vaccine that can recapitulate this lengthy process, infusion of bnAbs isolated from infected individuals has been proposed as an alternative approach for the prevention of HIV-1 acquisition.^2,3^

Among the HIV bnAbs isolated so far, those targeting the conserved MPER sequence typically display the desired breadth (i.e., they show nearly pan-neutralization) but have a moderate potency that limits their general application in therapy (IC_50_-*s* ≥ 0.1 μg mL^–1^).^3^ The anti-MPER bnAb 10E8 has emerged as a potential lead for optimization through engineering^4−8^ due to its relatively high neutralization potency, limited polyreactivity, and capacity to confer cross protection *in vivo* in primate models.^4,9−12^ Recent multispecific Ab platforms that have been engineered to simultaneously engage independent Env determinants (polyvalence) typically include 10E8 specificity in their designs.^5,13,14^ More recently, predictive modeling identified triple combinations including 10E8 specificity as having the highest coverage against currently circulating clade B viruses.^15^

The bnAb 10E8 binds to the highly conserved C-terminal subregion of MPER (ctMPER), which appears to fold as a continuous α-helix connecting MPER and TMD in the native Env.^16,17^ Together with Env surfaces, the lipid envelope constitutes a relevant component of the full “quinary” ctMPER epitope.^16−21^ The composition and structure of the lipid envelope are not subject to alteration through the genetic diversification processes that are at the basis of viral escape mechanisms. Therefore, elucidating at the nanoscopic level the functional role of bnAb interactions with this conserved element is key to devise new strategies for the optimization of ctMPER bnAbs^7,8,22^ and development of MPER-targeting vaccines.^17,20,23,24^

In recent work, we demonstrated that chemical modification of the Fab area that accommodates the membrane leads to higher affinity and enhanced avidity of Abs like 10E8, while preserving their neutralization breadth.^25,26^ The synthetic compounds utilized in that work were selected based on the high affinity of aromatic molecules for membrane interfaces.^27,28^ Thus, the analysis of the membrane interactions of chemically modified 10E8 Fabs could provide insights into the changes that occur in the lipid nanoenvironment surrounding MPER during the course of the neutralization process. Here, we investigate this issue computationally and experimentally using 10E8 Fabs subjected to targeted chemical modification (TCM) with bulky aromatics^25^ and VL surrogates of the complex viral membrane.^29^ The gathered evidence supports a correlation between the neutralization function, sorting of lipids with small headgroups, and the formation of a nonbilayer lipid nanoenvironment at the Fab-membrane interface. Together with the recruitment of sphingomyelin to the phospholipid-binding site of the Fab, this local deformation of the viral membrane may enhance the specific recognition of ctMPER helix. Elucidating the role of specific lipid types in the HIV neutralization process may help define new MPER peptide-liposome formulations that elicit stronger responses against the conserved ctMPER epitope. Additionally, the data support the application of the TCM method for optimizing antibodies targeting MPER-like epitopes and suggest pathways for the future development of this approach.

## 2 Results and Discussion

### 2.1 Calculations of the Free Energy Profiles of Permeation through a Viral-Like Lipid Bilayer

**Figure 1:**
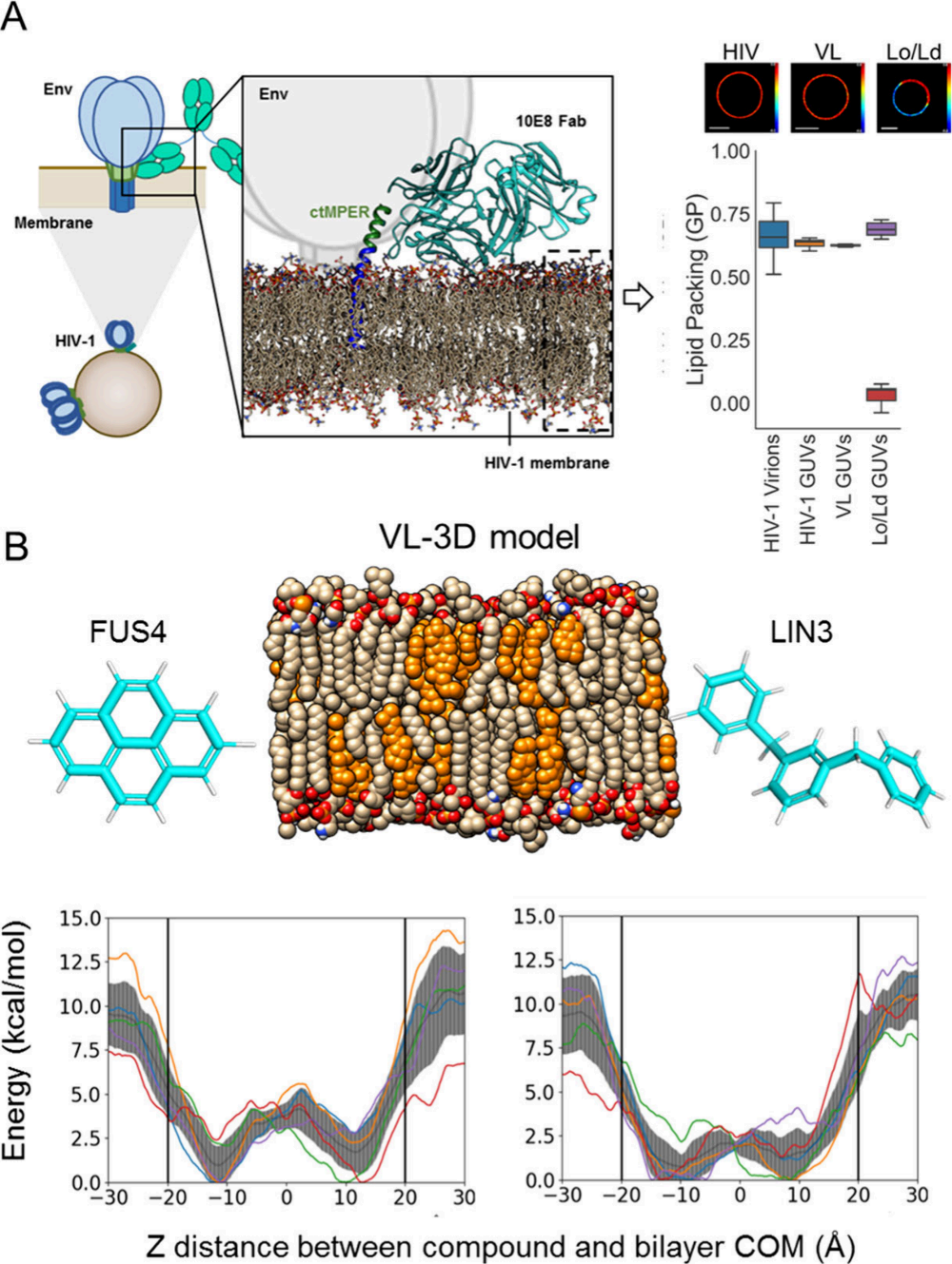
Potentials of mean force for the permeation process of Fus4 and Lin3 through the complex Viral-Like lipid bilayer. A) Quaternary structure of the 10E8 MPER epitope and VL membrane designation. The C-ter helix of MPER drives specific Ab binding, whereas the Fab accommodates surfaces contributed by the Env glycoprotein complex and the viral membrane. The panel on the right compares Laurdan General Polarization values measured in GUVs made of the VL mixture POPC:POPE:SPM:POPS:Chol (14:16:17:7:46 mol ratio), with those measured in GUVs made of lipids extracted from infectious virus^29^ (see also images on top) and those directly measured in virions.^31^ GUVs undergoing Lo/Ld phase separation are shown on the right. GUV images adapted from Huarte et al. (ref.^29^); available under a CC-BY 4.0 license; Copyright © 2016, The Author(s) B) Top: Structures of the VL bilayer model and free forms of Fus4 (pyrene) and Lin3 (1,4-dyphenilbenzene). Bottom: potentials of mean force (PMFs) from well-tempered metadynamics for the free permeants Fus4 and Lin3 molecules (left and right panels, respectively). In each case, five individual potential of mean force (PMF) profiles for each compound were calculated to account for the complexity of the lipid bilayer by placing the small molecules at different xy positions on the membrane at the starting point; each colored line corresponds to a single well-tempered metadynamics simulation starting with the compound located at different xy positions of the bilayer. The PMFs cannot be identical as the membrane is not homogeneous, and it will vary depending on the composition at the initial xy position. The shaded areas indicate the average values from the five independent simulations.

The structural model displayed in Figure 1A depicts the full quaternary 10E8 epitope as consisting of a section of the continuous MPER-TMD helix, viral membrane lipids, and a surface derived from the Env ectodomain.^16^ On the basis of bilayer lipid packing quantification in single vesicles, we established in previous work a viral-like (VL) synthetic lipid mixture of composition POPC:POPE:SPM:POPS:Chol (14:16:17:7:46 mol ratio).^29,30^ The lipid packing degree measured in VL-GUVs was in the range of that measured for segregated Lo domains, and matched that of GUVs made from lipids extracted from purified infectious virions,^29^ and also that measured directly in virions^31^ (Figure 1A, right panel). Thus, to analyze interactions of Fabs chemically modified with aromatics with the HIV lipid envelope, we built a VL-based surrogate of this complex membrane using the CHARMM-GUI^32^ membrane builder module (Figure 1B, top). The system was then solvated and neutralized using a 0.15 M KCl solution resulting in a simulation box of 170 × 170 × 160 Å^3^ dimensions.

Among the series of synthetic aromatic compounds tested, those featuring a phenyl moiety linked via a flexible spacer (Lin3) and a polycyclic aromatic compound with a pyrenyl group (Fus4) were the most effective at enhancing the functional activity of MPER bnAbs when attached to Fab surfaces contacting the HIV membrane.^25^ In addition, the length and flexibility of Lin3 together with the chemical nature of its constituent phenyl rings contrast with the compactness and rigidity of Fus4 membered rings (Figure 1B, top), potentially giving rise to different interaction modes and preferential locations within the viral envelope bilayer, akin to trends found after comparing the aromatic side chains Phe and Trp.^28^ Hence, we selected Lin3 and Fus4 for further computational analyses (Table 1).

**Table 1:** Summary of Simulations Considered in This Study

**Notation**	**Membrane Composition**	**Simulation Time (μs)**^a^	**Computational Method**
Fus4	COMPLEX Virus like	0.96	Classical MD
Lin3		0.95	Flooding
Fus4		1.35	Parallel Tempering Metadynamics
Lin3		1.48	
S65YCMFus4 + TM		3 × 1.0	Classical MD
S65YCMLin3 + TM		3 × 1.0	

a TOTAL Simulation Time: ∼ 10 μs.

To compare their membrane affinity and stability, we first established the free energy profiles of permeation of these two aromatic molecules through the viral membrane (Figure 1B, bottom). Thus, we determined the potentials of mean force (PMFs) from well-tempered metadynamics for the entire permeation process through the complex VL mixture of each molecule when moving from the aqueous solution to the bilayer center and from the bilayer center to the internal milieu. We repeated this process five times to take into consideration the complexity of the VL bilayer with starting points at different positions on the x-plane of the membrane. The shape of the PMF has values of Δ*G*(|z|) that first decrease and then increase toward the bilayer center, with the central barrier along |z| relatively small.

The initial free energy barrier along the axis perpendicular to the bilayer plane, |z|, corresponds to Fus4/Lin3 interactions with lipid headgroups, and the second one corresponds to the bilayer center, which is lower relative to the bulk solution. The maximum values in the PMF are 9.8 ± 2.5 and 9.8 ± 1.4 kcal/mol, respectively, for Fus4 and Lin3. As the bilayer is not exactly symmetrical, the maximum values starting from the opposite leaflet rendered similar but not identical values of 8.5 ± 1.9 and 8.8 ± 2.1 kcal/mol for Fus4 and Lin3, respectively. The maximum energy barrier that Fus4 must overcome to cross the center of the bilayer was found to be 3.1 ± 1.1 kcal/mol while for Lin3, it was relatively lower at 1.3 ± 0.9 kcal/mol.

### 2.2 Binding to Single Vesicles by Quantitative Microscopy

**Figure 2:**
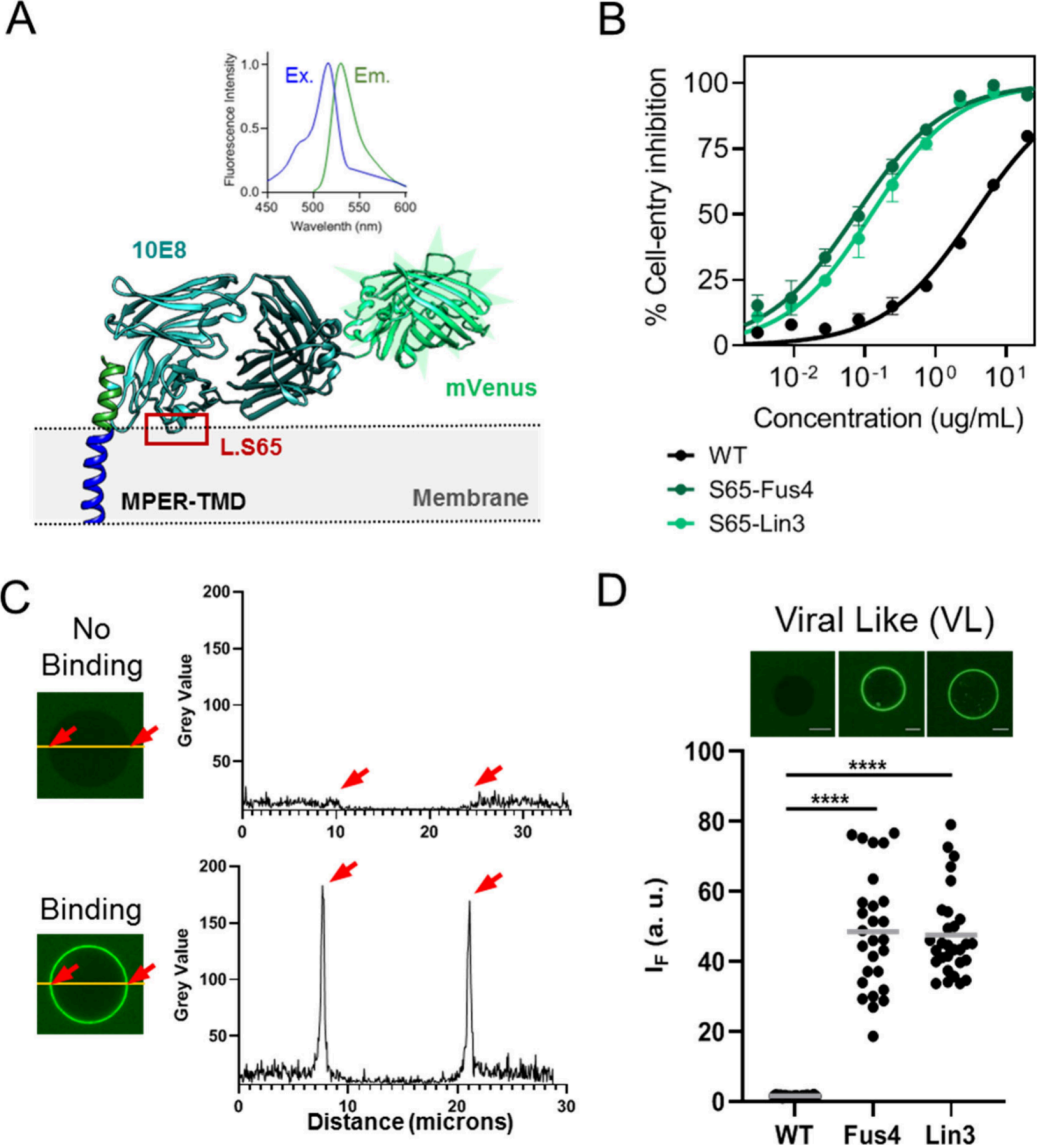
Binding to single VL vesicles using Fab-mVenus chimeras. A) Structure depicting the 10E8 Fab-mVenus chimera bound to the ctMPER-TMD helix. Excitation/Emission spectra of the purified protein are shown on top, demonstrating the acquisition of the correct tertiary structure by mVenus. B) Cell-entry inhibition activity comparing WT and chemically modified chimeras. Titration values are means ± SD of three independent experiments. C) Quantification of Fab 10E8 binding to single vesicles using the Fab-mVenus chimera. Left panels display confocal microscopy images of single VL vesicles incubated with Fab-mVenus WT or Fab-mVenus-Lin3 (top and bottom, respectively). Traces on the right panels follow the changes in the mVenus fluorescence intensity at the equatorial plane (green label). D) Binding to single VL vesicles comparing the WT chimera with those chemically modified with Fus4 or Lin3. Amount of Fab bound was estimated for each vesicle as the fold increase in mVenus fluorescence intensity over the mean value of the background level (i.e., background intensity normalized to 1). (****p < 0.0001).

To test experimentally affinities for VL membranes, we next measured binding to single vesicles by quantitative fluorescence microscopy. Previous analyses of the 10E8 binding function involved the use of iodoacetamide derivatized with fluorescent probes for their conjugation at defined Fab positions.^7,33^ However, the requirement of simultaneous conjugation of fluorescent probes and synthetic aromatics at defined sites hindered TCM following this method. As an alternative approach, we considered fusing the 10E8 HC to the fluorescent mVenus protein, which, upon coexpression with the LC and Fab assembly, would allow TCM of its FRL3 (Figure 2A). Pseudovirus-based neutralization assays confirmed that labeling of the FRL3 with Fus4 or Lin3 also improved the antiviral potency of Fab-mVenus (Figure 2B), thus validating functionally this fluorescent chimera for subsequent binding analyses (Figure 2C).

As expected from the absence of lipid polyreactivity reported for the antibody 10E8,^4,6^ the unmodified Fab did not bind to VL GUVs (Figure 2D). In contrast, chemical modification with Fus4 or Lin3 resulted in substantial binding of Fab-mVenus to the VL membranes. Therefore, the experimental evidence seems to confirm that the strong tendency for insertion of the compounds conferred to the Fab the capacity for interacting with VL membranes.

**Figure 3:**
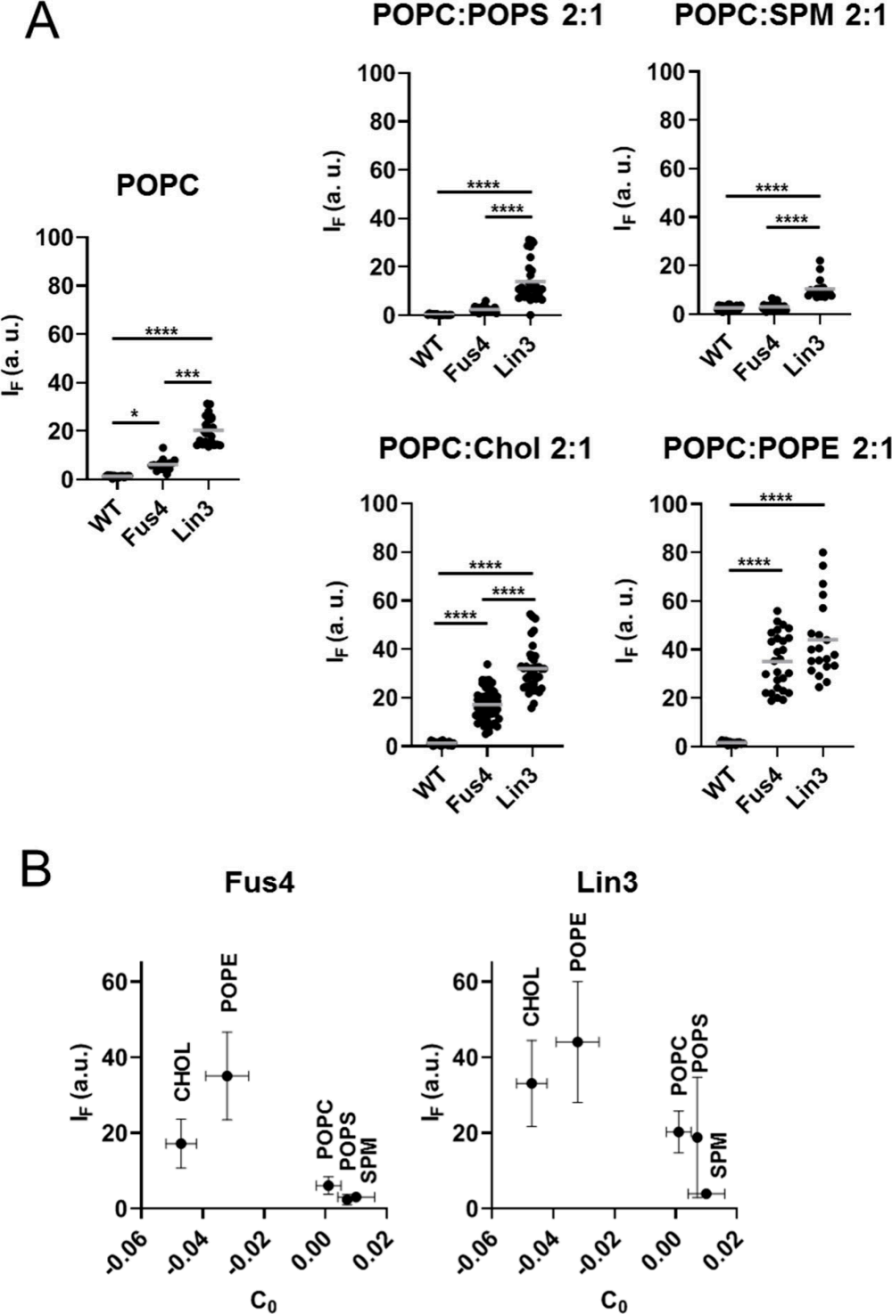
Effect of single components of the VL mixture on Fab-membrane binding. A) GUVs made of pure POPC were compared with mixtures containing 20 mol % single VL lipids as indicated in the panels. Conditions otherwise as in previous Figure 2D. (****p < 0.0001, ***p < 0.0002, *p < 0.03). B) Dependence of Fab-membrane binding on the spontaneous curvatures (C_0_-s) of the added lipids. C_0_ values (means ± SD) were obtained from reference.^34^

With the aim of identifying in the complex mixture the lipids that conferred affinity for VL membranes, we next mixed SPM, POPS, POPE or Chol with a constant proportion of POPC and determined binding extents by quantitative microscopy (Figure 3A). For the Fabs modified with Lin3, the data indicated a certain level of polyreactivity with POPC membranes that diminished progressively in the presence of POPS and SPM. In contrast, Fab-Lin3 binding appeared to increase in mixtures containing Chol or POPE. Fabs conjugated to Fus4 followed a similar trend but were overall less polyreactive.

These observations appear to indicate that association-insertion into lipid bilayers of the chemically modified Fab 10E8 would be facilitated by lipids of the VL membrane with a negative spontaneous curvature (C_0_), whereas those displaying positive C_0_ would oppose this effect (Figure 3B). We further tested this possibility using the archetypical nonbilayer lipids diacylglycerol (DAG) and lysophosphatidylcholine (LPC) with negative (−0.087) and positive (+0.026) C_0_-s, respectively.^34^ Addition of 10 mol % DAG already promoted the spontaneous insertion of Fab-Lin3 into POPC bilayers, whereas inclusion of the same proportion of LPC blocked the process (Figure S1). Thus, the Fab 10E8 subjected to TCM seems to behave as a peripheral membrane protein that inserts favorably into hydrophobic interfacial sites created by the small headgroups of nonbilayer lipids.^35,36^

### 2.3 Binding of Chemically Modified Fabs to Antigen-Expressing Cells and Upgraded Membrane Models

**Figure 4:**
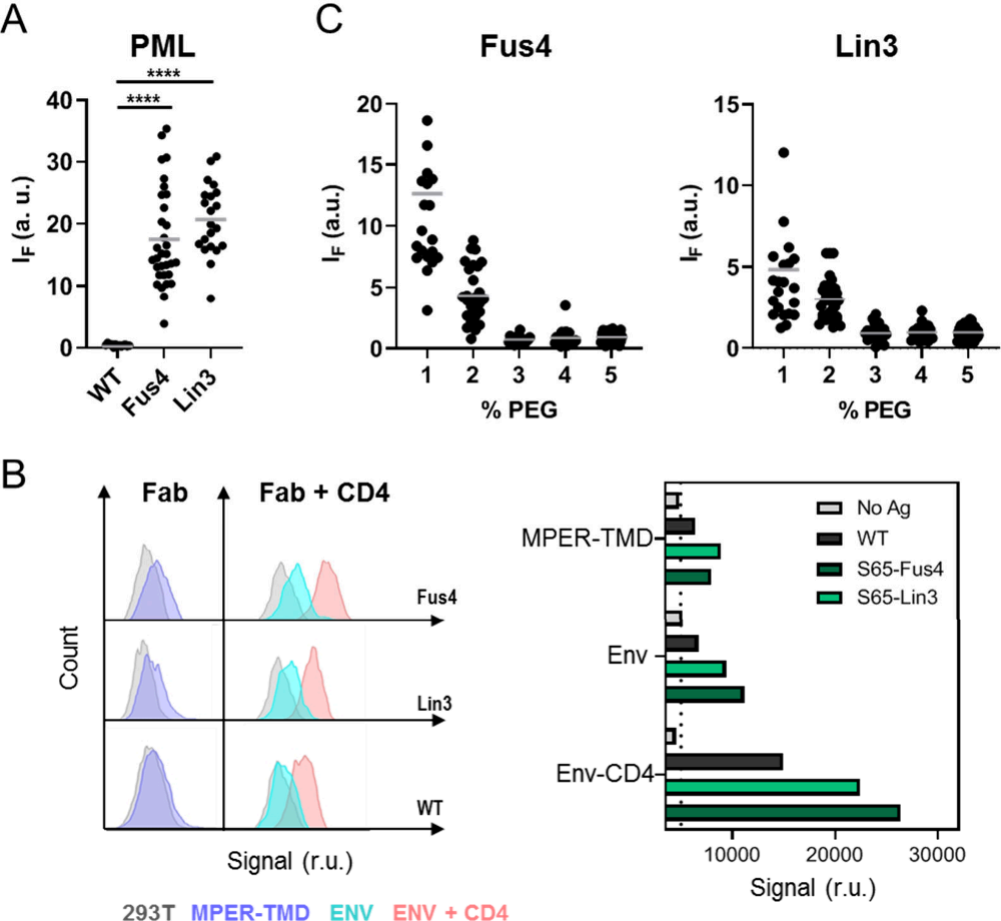
Binding of chemically modified Fab-mVenus chimera to PML GUVs and antigen-expressing cells. A) Binding of Fab-mVenus chimeras to PML vesicles made of POPC:Chol:SPM (40:40:10 mol ratio) emulating the exofacial plasma membrane leaflet.^44^ Conditions are otherwise as described in Figure 2D. (****p < 0.0001). B) Flow cytometry analysis of cells displaying MPER antigens and stained using the WT 10E8 Fab-mVenus chimera and its chemically modified versions. Histograms compare cells expressing MPER-TMD (left) with cells expressing HIV Env ADA.CM.v4 (right) in the presence and absence of soluble CD4 (red and blue histograms, respectively). In both instances gray histograms correspond to the background signal of untreated cells. C) Effect of increasing concentrations of PEG–PE on Fab-mVenus binding to PML vesicles.

The composition of the HIV-1 lipidome suggests that the viral envelope is acquired from nanodomains containing high amounts of Chol and SPM,^37^ akin in composition to the external monolayer of eukaryotic cell plasma membranes.^38−40^ Thus, we inferred that TCM could also boost molecular recognition of antigens at the cell plasma membrane following similar mechanisms. As shown in Figure 4A, conjugation with the compounds also resulted in efficient association of the Fab-mVenus with plasma membrane-like (PML) GUVs.

Fluorescent Fab-mVenus variants were tested for binding to antigen-expressing cells using flow cytometry (Figures 4B and S2). A modest increase of mVenus fluorescence intensity over the background level was observed upon incubation of the unmodified Fab-mVenus with cells expressing constitutively the MPER-TMD polypeptide.^41^ The intensity of the mVenus signal increased markedly upon conjugation of the relevant 10E8 moiety with either Fus4 or Lin3, consistent with more efficient binding upon chemical modification of the Fab. Binding improvement after TCM could be more clearly discerned when Fab-mVenus fusions were incubated with cells expressing ADA.CM.v4 Env glycoprotein.^41,42^ Preincubation of these cells with mD1.22, an optimized, soluble form of the CD4 D1 domain,^43^ increased the amount of Fab-mVenus associated with cells and, under these conditions, a substantially higher binding capacity of the Fab-mVenus was also observed with the Fus4 or Lin3 TCM variants of 10E8.

In summary, TCM of 10E8 using the aromatic compounds improved specific antigen recognition on the surface of cells expressing MPER in different formats, including the N-terminal extremity of the MPER-TMD construct; in the context of the prefusion Env complex; and upon CD4-induced activation, a condition that has been shown to increase MPER accessibility, and enhance antibody binding.^41^ Thus, the level of binding enhancement promoted by TCM, increased in consonance with the degree of MPER accessibility. Notably, following incubation of 10E8 with cells that did not express any form of MPER antigen, cell staining by the fluorescent Fabs, whether unlabeled or labeled with Fus4 or Lin3, was in the range of the background staining of cells incubated in the absence of antibody. Together, these observations are consistent with the highly limited spontaneous interactions with the cell plasma membrane and, hence, with the strict dependence of binding on antigen presentation, a phenomenon that was improved by the TCM of the Fabs.

To explain the discrepancy between cell and GUV results (Figures 4A and B, respectively), we considered a key feature of the cell plasma membrane that was not reproduced by the model bilayers; that is, the exofacial monolayer is covered by a crowded-hydrophilic carbohydrate layer, which might hinder antibody accessibility to hydrophobic bilayer patches. Thus, we mimicked this hydrophilic layer, by including lipids in the membrane composition with polyethylene glycerol (PEG) covalently attached, as previously described.^45,46^ Inclusion of chemically inert PEG–PE in the range of 1–5 mol % is predicted to efficiently cover the GUV surface with a highly hydrated, continuous layer of this voluminous polymer, and to reduce nonspecific protein adsorption onto the lipid bilayer.^47,48^ PML GUVs that contained PEG–PE within this concentration range were found to bind progressively lower amounts of the chemically modified Fab-mVenus, and they did so until they reached the background levels displayed by the unmodified version (Figure 4C).

### 2.4 Binding to ctMPER Reconstituted in Vesicles by Quantitative Microscopy

**Figure 5:**
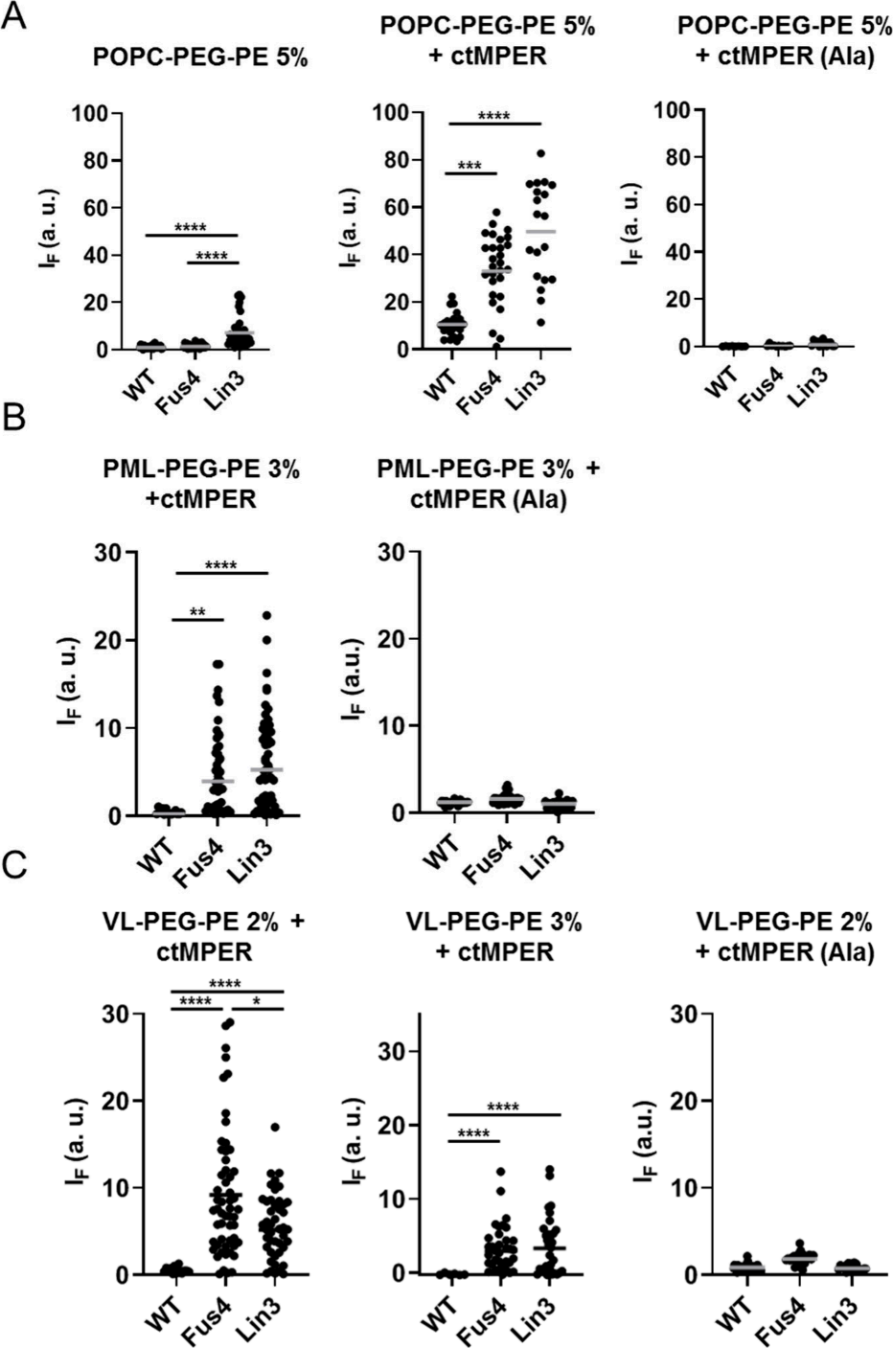
Effect of chemical modification on specific binding to the ctMPER epitope reconstituted in membranes. A) Binding to POPC:PEG–PE (95:5 mol ratio) vesicles was measured in the absence (left panel) or presence of ctMPER-TMD or ctMPER-TMD(Ala) peptides reconstituted in membranes (center and right panels, respectively). The peptides were included at a 1:250 peptide-to-lipid ratio (mol:mol). B) Binding to PML vesicles including 3 mol % PEG–PE to avoid off-target spontaneous partitioning into membranes (see Figure 4). C) Binding to VL vesicles including 2 or 3 mol % PEG–PE. (****p < 0.0001, ***p < 0.0002, **p < 0.02, *p < 0.03).

The cell data above suggested that the presence of a hydrophilic carbohydrate layer influences the specific recognition of the ctMPER epitope and that TCM can elicit binding to Env antigen in the absence of spontaneous association with the plasma membrane. Thus, we hypothesized that once the Fab-ctMPER complex forms, the lipid nanoenvironment can regulate bnAb activity following mechanisms analogous to those described for integral membrane proteins.^49^ To obtain evidence supporting this assumption, we reconstituted the ctMPER-TMD peptide (Figure S3) in PEGylated GUVs, so that Fab binding in this system evolved in the absence of spontaneous interactions with lipids. Specific recognition ctMPER-TMD was first assessed in POPC lipid bilayers, a general model for cell membranes (Figure 5A). Supporting the occurrence of an epitope-dependent, specific binding phenomenon, the three fluorescent chimeras bound to POPC:PEG–PE (95:5, mol:mol) GUVs that presented the ctMPER epitope on their surface, but not to those devoid of peptide, nor to those that presented ctMPER (Ala), a variant with the critical residues for epitope recognition 672WF673,^4,33^ substituted with Ala (Figure S3). Moreover, TCM with Fus4 or Lin3 improved specific binding to GUVs that contained the wt ctMPER-TMD sequence, in line with the observations previously made in the cell system.

In the PML and VL surrogates, the effect of TCM was even more strikingly evident (Figures 5B and C, respectively). The unmodified Fab did not bind to PEGylated PML or VL GUVs, not even in the presence of the reconstituted ctMPER-TMD epitope peptide, consistent with the restricted binding to MPER epitope peptides reported to occur in Chol-rich thick/rigid bilayers^44,50^ (see also Figure S4). Modification with Fus4 or Lin3 circumvented this restriction and restored the capacity of the Fab for binding to the PEGylated GUVs that contained ctMPER-TMD, but not to those containing ctMPER-TMD(Ala). Thus, in the Chol-enriched surrogates of the cell plasma and viral membranes, TCM boosted the specific binding of the Fab to GUVs that contained the 10E8 epitope peptide.

### 2.5 Lipid Nanoenvironment from Atomistic MD Simulations

**Figure 6:**
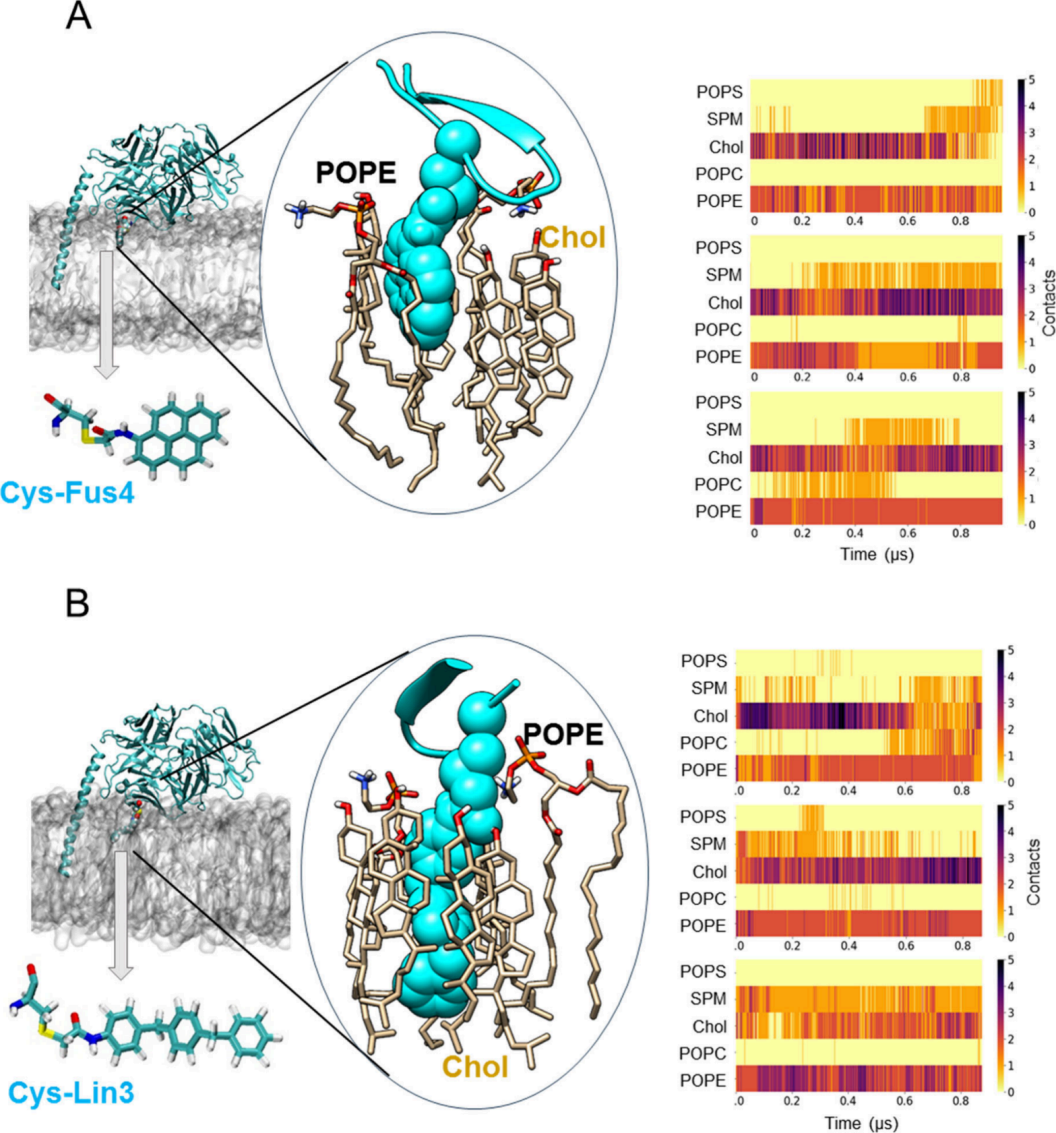
Lipid contacts elucidated from MD simulations of Fab-ctMPER-TMD complexes inserted into VL bilayers. Representative snapshots of the Fabs with a chemical modification engineered to contain a single Cys residue at position 65 within the Fab’s FRL3 to which (A) Fus4 and (B) Lin3 were attached in the presence of the MPER-TMD inserted in the viral-like membrane. The protein is shown in cyan using a cartoon representation, and the point mutations are displayed as van der Waals spheres within the snapshot. Cys-Fus4 and Cys-Lin3 are also depicted separately in the licorice representation. A zoomed-in view highlights some of the lipid–protein interactions observed, which are established with Cys-Fus4 and Cys-Lin3 represented in van der Waals in cyan. The right panels show the evolution with simulation time of the number of contacts between each lipid component of the viral-like membrane and each single mutated residue in the antibody, either Cys-Fus4 or Cys-Lin3. A contact is considered when the distance between any heavy atom of the mutated residue and any heavy atom of a lipid residue is ≤3.5 Å.

To get insights into the lipid nanoenvironment upon formation of the Fab-ctMPER complex, we ran MD simulations of Fab-ctMPER-TMD complexes implanted into the VL lipid bilayer. The starting model for the simulations was the X-ray crystal structure at 2.40 Å resolution of the Fab 10E8 with an elongated epitope peptide bound (PDB ID 5GHW).^18^ The peptide comprises a continuous helix spanning the gp41 MPER-TMD junction, including residues 671–687. The rest of the TMD moiety until residue R709 was modeled as in the crystal structure of the Fab LN01 in complex with a similar ctMPER-TMD peptide (PDB ID 6SNE).^51^ The Fab-peptide orientation observed in the crystal structure of the Fab 10E8 with respect to the MPER-TMD helix was preserved in this configuration.^18^ Following the experimental protocol, a chemical modification was first engineered to contain a single Cys residue at position 65 within the Fab’s FRL3, and subsequently, Fus4 or Lin3 were linked via a flexible spacer. Left panels in Figures 6A and B respectively display snapshots of the Fabs modified with Fus4 or Lin3 (top) and the compounds derivatized with Ser as modeled for running the simulations (bottom).

We first analyzed in three different simulations the lipid nanoenvironment of Fus 4 in the VL mixture (Figure 6A, center and right panels). The center panel depicts the compound surrounded by Chol and POPE molecules at a given time in the simulation. Determination of the number of contacts reflected a preferential interaction with Chol and POPE (right panel and Table 2). Similar lipid interaction profiles were observed for the Lin3 compound derivatized with Fab (Figure 6B and Table 2). In both instances, the third most prominent interaction of the compounds in the simulations appeared to be established with SPM (Table 2).

**Table 2:** Average Number of Contacts between Each Lipid Component of the VL Mixture and (i) the Chemically Modified Residue in the Antibody, In the Three Replicas Considered Per System, Or Contacts between Each Lipid Component and (ii) Lin3 or Fus4 Free Molecules in Simulations Where 25 of Them Were Present, and the Maximum Number of Molecules Binding Each Lipid on a Per Frame Basis Was Averaged. The Composition of the VL Bilayer Used Is Indicated in the First and Second Columns

**Lipid**	**Mol%**	**(i) Lin3**	**(i) Fus4**	**(ii) Lin3**	**(ii) Fus4**
POPS	7	0.0 ± 0.0	0.0 ± 0.1	2.2 ± 0.7	1.7 ± 0.6
SPM	17	0.7 ± 0.4	0.4 ± 0.5	2.6 ± 0.6	2.4 ± 0.6
Chol	46	2.3 ± 0.7	2.5 ± 0.9	4.2 ± 1.0	3.7 ± 0.7
POPC	14	0.2 ± 0.4	0.1 ± 0.3	3.5 ± 0.8	2.4 ± 0.5
POPE	16	2.1 ± 0.3	1.8 ± 0.5	3.3 ± 0.8	2.6 ± 0.6

These preferential contacts might be reflecting the chemical affinity of the bulky aromatics for these lipids of the VL mixture. To test this possibility, we also computed the number of interactions established by the free forms of Fus4 and Lin3 with the different VL lipids (Figure S5A). In contrast to the compounds derivatized with Fab, the number of contacts established by the free forms embedded in the VL bilayer was closely proportional to the lipid mole ratios (Table 2 and Figure, S5B). Thus, the number of recorded contacts appeared to reflect the probability of collision with the components of the VL hydrocarbon-core and, therefore, to rule out the establishment of long-lasting interactions with any of them.

**Figure 7:**
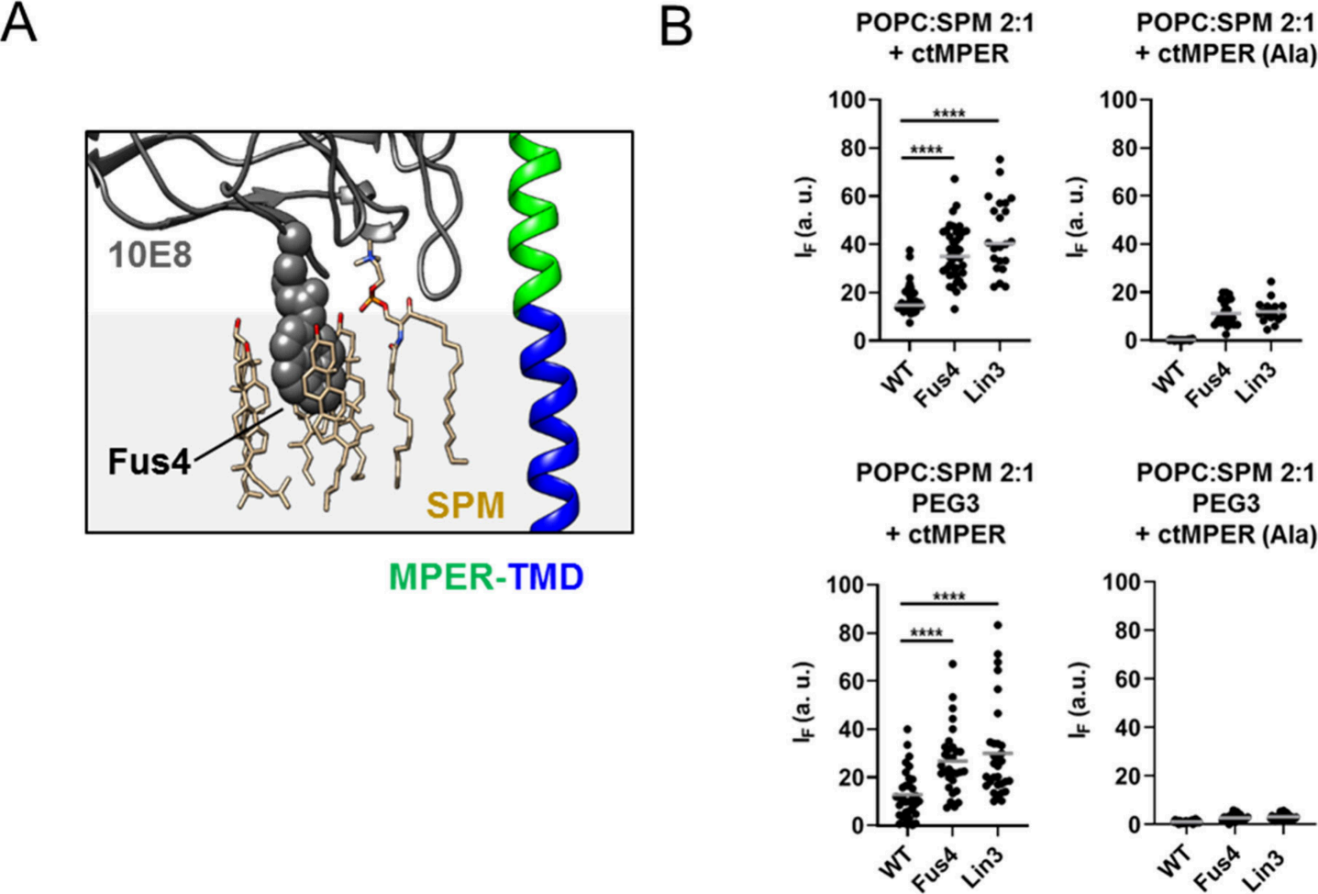
Sphingomyelin binding to the phospholipid binding site of 10E8. A) Snapshot displaying an SPM molecule bound to the PL-binding site of 10E8, located in close proximity to Chol molecules gathered around Fus4. B) Binding to GUV-s made of POPC:SPM (2:1) that contained the ctMPER-TMD or ctMPER-TMD (Ala) peptides reconstituted (left and right panels, respectively) in the absence or presence of PEG–PE (top and bottom panels, respectively). (****p < 0.0001).

In conclusion, the above MD simulations identify Chol and POPE as the VL components that preferentially interact with the elements of the Fab 10E8 that accommodate the membrane upon interaction with the ctMPER epitope. In addition, in the membrane-inserted Fab-ctMPER-TMD complex the SPM molecules that are close to the compounds appeared to occupy the phospholipid binding site configured by the CDRL1, FRL3 and CDRH3 elements of the Fab^19^ (Figure 7A). However, although SPM predominantly occupied this site, the Fab 10E8 in solution did not show any relevant affinity for SPM when presented as a component of a lipid bilayer (see Figure 3A). Thus, binding processes scored in the POPC:SPM (2:1) mixture were closely selective for the ctMPER-TMD-containing GUVs, even in the absence of a PEG carbohydrate layer (Figure 7B). In this setting, the TCM also conferred higher capacity to the Fabs for MPER specific binding.

### 2.6 Model for the Effects of the Lipid Nanoenvironment on the Mechanism Of ctMPER Molecular Recognition

The surrounding lipid nanoenvironment regulates the structure and function of integral membrane proteins, either by defining collective properties as thickness, packing or intrinsic curvature, and/or by establishing specific interactions with lipid molecules.^49^ Here, we hypothesized that similarly the HIV neutralization function of ctMPER-targeting bnAbs could be regulated by the chemically complex viral membrane at the site where the formation of the Fab-ctMPER complex takes place. To approach the relationship between the neutralization function of 10E8 and the lipid nanoenvironment surrounding the ctMPER-Fab complex, we used Fabs potentiated through TCM with Fus4 or Lin3.^25^ To confirm the affinity of Fus4 and Lin3 for the viral membrane, we first determined for each molecule the PMF for the entire permeation process through the complex VL mixture that emulates the lipid packing conditions of the viral envelope.^29,31^ In the VL system, the energy needed to extract Fus4 or Lin3 from the bilayer hydrocarbon core to the water interface is similar and estimated to be in the range of 10 kcal mol^–1^ (i.e., corresponding to *K*_p_-s in the order of 10^7^). Thus, the TCM with either Fus4 or Lin3 may theoretically confer to Fabs high affinity for VL membranes. The quantitative microscopy studies using a fluorescent Fab-mVenus chimera and VL GUVs gave support to this idea and allowed the identification of the contribution of individual lipids to the process.

**Figure 8:**
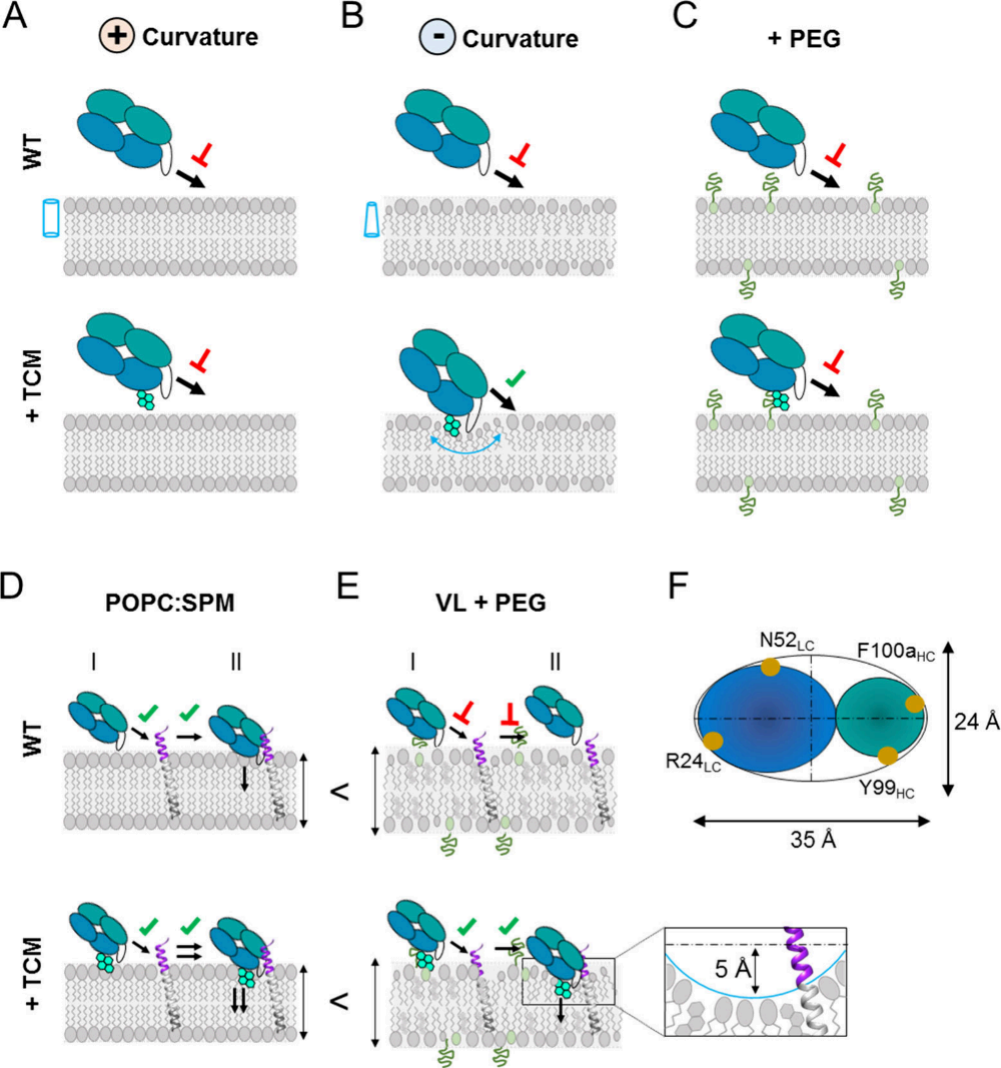
Model for the recognition of membrane lipids and ctMPER by Fab 10E8 and effects of the lipid nanoenvironment in the process. A) 10E8 does not bind spontaneously to membranes made of lipids with positive spontaneous curvature (cylinder) (top), not even after being subjected to TCM (bottom). B) Inclusion of lipids with negative spontaneous curvature (truncated cones) does not affect the Fab WT (top) but facilitates direct access of chemically modified Fabs to membrane lipids (bottom). C) Incorporation of a hydrocarbon layer (PEG) precludes direct access of Fabs to membrane lipids. D) Reconstitution of the ctMPER epitope in membranes made of lipids with positive spontaneous curvature sustains the specific binding of Fab WT and its chemically modified variants (top and bottom panels, respectively). However, TCM enhances Fab affinity for ctMPER suggesting the occurrence of a two-step mechanism in this system: (I) encounter and (II) docking into the membrane. TCM would specifically promote membrane insertion after the initial recognition. E) Model for the compound-mediated specific recognition of ctMPER by Fab 10E8 in the thick-rigid VL membrane. In this system, the WT Fab does not associate with membranes, not even upon inclusion of the ctMPER-TMD peptide (top). TCM may boost the specific recognition of ctMPER-TMD by sorting lipids with small polar headgroups and locally generating a membrane deformation (bottom). The model proposes a depth of 5 Å for the deformation based on previous atomic force microscopy studies.^30^ In addition, its surface is assumed to be an ellipsoid (F panel).

In those experiments, interaction of Fabs subjected to TCM with VL GUVs was promoted by POPE and Chol, two lipids with negative spontaneous curvatures,^34^ whereas POPS and SPM, two lipids with slightly positive curvature seemed to restrain the process. The models depicted in Figures 8A,B illustrate these findings. Fabs modified with Fus4/Lin3 could insert efficiently into model membranes devoid of the ctMPER epitope but only in the presence of nonbilayer lipids with negative curvatures. It has been argued that due to their small size, a reduction of the bilayer lateral pressure at the headgroup level can create hydrophobic interfacial sites, which facilitate protein insertion.^35,52^

Experimental binding also revealed that TCM could generate comparable Fab affinities toward PML GUVs with a Chol-enriched lipid composition based on the exofacial monolayer of the cell plasma membrane. However, the chemically modified Fabs showed limited spontaneous interaction with the membranes of cultured cells. We surmise that access to interfacial insertion sites was hindered in cells by the hydrocarbon layer projecting from the external plasma membrane leaflet, and that this condition could be reproduced in PML models by the inclusion of PEG–PE into the lipid mixture (Figure 8C). Importantly, Fus4 and Lin3 compounds enhanced ctMPER recognition in antigen-expressing cells and PML model membranes, the latter upgraded by incorporating a hydrocarbon layer that precluded the spontaneous partitioning of the chemically modified Fabs. Thus, the results in cells and PEGylated models support an antigen-dependent recruitment of Fabs to the membrane environment. Under these conditions, TCM increases overall binding affinity for the ctMPER-TMD sequence reconstituted in the membrane. Attending to this evidence, we infer that contribution of individual lipids to the binding strength and neutralization occurred after, or concomitantly to, Fab engagement with the ctMPER epitope (model in Figure 8D).

PEGylation also prevented the spontaneous binding of Fabs to the VL membranes. In these upgraded models of the viral envelope, TCM was required to boost the specific epitope recognition. As proposed in the model depicted in Figure 8E (top), the increased thickness of the Chol-enriched VL membrane may restrict the accessibility to the ctMPER epitope at the interface (I encounter), whereas its stiffness may act against the accommodation of the Fab onto its surface (II docking). Fabs modified with Fus4 and Lin3 were able to circumvent this restriction and gain access to the antigen for specific binding.

The mechanism underlying this postbinding effect was analyzed in all-atom MD simulations of Fabs bound to a ctMPER-TMD helix immersed in the VL bilayer. The simulations revealed that the obstacle imposed by stiffness and thickness on ctMPER binding can be partially overcome by the compounds derivatized with Fab. The force exerted by the compounds at the Fab-membrane contact appears to induce sorting of the VL components with the smallest headgroups, POPE and Chol. In this nanoenvironment, a deformation of the monolayer can be created that facilitates access to the ctMPER helix and the subtle insertion (accommodation) of the Fab into the membrane interface (Figure 8E, bottom). To sustain this possibility, at least theoretically, we may first approximate the amount of energy opposing the Fab-induced deformation of the VL monolayer, either by calculating the energy of bending, (Δ*G*_*c*_), or based on estimates of the Young’s compressibility modulus (*E*), and then compare its magnitude with the force that a single compound can exert.

In the first case, Δ*G*_*c*_ can be approximated from the area of the deformation (*A*) (Figure 8F), the monolayer bending modulus (*κ*_m_) and the difference of the curvatures in the deformation (*c*_1_ + *c*_2_) with respect to the spontaneous curvature in absence of any stress (*c*_0_), as given by [Δ*G*_*c*_ = (1/2)·*A* κ_m_·(*c*_1_ + *c*_2_ - *c*_0_)^2^] (ref.^34^). The curvatures *c*_1_ and *c*_2_ can be derived from the geometry of the deformation (Figures 8F and S6). Using a generic value for *κ*_m_ ≈ 10*k*_B_*T*^53^ and a *c*_0_ ≈ −0.02 determined for a Chol-enriched ordered domain,^54^ Δ*G*_*c*_ would amount to ca. 0.65 × 10^–19^ J. However, values of *κ*_m_ as high as 40*k*_B_*T* have been reported for POPC-based mixtures containing high proportions of Chol.^55^ Therefore, one might expect that a maximum value of Δ*G*_*c*_ could be in the range of 2.4 × 10^–19^ J, but not much higher. In the second case, an *E* ≈ 140 MPa is generally assumed as determined by Force spectroscopy for laterally segregated ordered domains,^56^ which would correspond to a resistance energy against monolayer deformation, *F*_m_, of ca. 4.6 × 10^–19^ J. Again, *E* values as low as ca. 10 MPa have been reported more recently in the literature,^57^ so it is reasonable to assume that *F*_m_ values could lie between 0.3 and 4.6 × 10^–19^ J.

These energy values are to be compared with the force that can theoretically exert a single compound, which can be estimated from the lowest energy value obtained in the depth-dependent analysis and thought to be in the order of 2.5 × 10^–18^ J. Thus, at least from the point of view of a continuous approach,^53^ Fus4 and Lin3 may contribute enough energy as to generate a negatively curved deformation in the VL monolayer for better accommodation of the Fab and optimal engagement with the ctMPER helix. At the nanoscopic scale, generation of this defect appears to involve the sorting of nonbilayer VL lipids to the Fab-membrane area of contact.

## 3 Conclusions

Our observations highlight the crucial role of the complex lipid nanoenvironment surrounding MPER in the functional activity of HIV-1 neutralizing antibodies. We infer important implications from this work for the future development of MPER therapeutic antibodies and vaccines. On the one hand, data recovered from the Antibody Mediated Prevention (AMP) efficacy trial^58^ reinforce the idea that bnAbs endowed with sufficiently high potency and breadth can become useful agents to prevent and treat infection by HIV-1. Subsequent isolation of HIV variants from the participants who acquired infection allowed establishing a combination of bnAbs, including 10E8, which would be effective against circulating clade B viruses.^15^ Thus, the prospective applications of 10E8 to immunotherapy warrant current efforts to improve its stability and potency.^6−8^

Based on the assumption that strengthening interactions with the membrane interface may increase Ab affinity for membrane-proximal protein epitopes, we proposed a general pathway for upgrading 10E8-like MPER Abs, namely, site-selective chemical modification with synthetic aromatic compounds^25^ (designated here as TCM). We primarily selected aromatic compounds, not only under the assumption that they would promote water-to-membrane Ab transfer (i.e., that they would provide the Fab surface with interfacial hydrophobicity) but also because we surmised that their partial polar-amphiphilic character would compromise to a lower extent the stability of the Ab in solution or in serum (when compared to other hydrophobic moieties as, for example, fatty acids). However, we can speculate on the potential use of saturated chains for compound optimization, for instance, as linkers to extend their structure or as alkyl substituents on aromatic rings to vary their polarity.

Despite this broader potential, constraints that might limit *in vivo* applications of TCM remained undefined. Most critically, the possible boosting of undesired off-target Ab binding to ubiquitous cell membrane lipids is a concern. Thus, to be useful, TCM should ideally promote interactions with the cell or viral membrane upon formation of the Fab-ctMPER complex, while avoiding nonspecific association with tissue cell membranes before engaging with the antigen. In this methodological context, our data establish that TCM enhances antigen binding after specific recognition of ctMPER. We conclude that future TCM development will likely require the establishment of Structure–Activity Relationship (SAR) taking this mechanism into account, i.e., screenings for more efficient compounds should be performed under conditions that allow the detection of binding strengthening after specific Fab engagement with the membrane-inserted epitope.

On the other hand, the recently ended HVTN 133 trial has confirmed the safety and immunogenicity of an MPER peptide/liposome vaccine in HIV-uninfected individuals.^17,24^ Moreover, the study has served to demonstrate the capacity of a synthetic vaccine to induce MPER B-cell lineages and select for functional improbable mutations, the latter required for the emergence of MPER-targeted neutralizing activity. However, serum Ab responses appeared to focus on the more variable N-terminal MPER subregion (ntMPER) and, hence, the isolated monoclonal Abs tended to display low breadth and potency.^24^ Despite these limitations, the outcome of this study warrants further efforts to design vaccines producing Abs that target the more conserved ctMPER section.

According to our data, an effective ctMPER-targeting vaccine designed to generate potent 10E8-like responses should elicit Abs by combining the specific binding to the ctMPER residues within the membrane-anchored MPER-TMD helix, with the establishment of ancillary interactions with lipids of the viral membrane, both events occurring sequentially. The vaccine evaluated in the HVTN 133 trial contained liposomes that were PEGylated to promote their stability in serum. Our results showed that the addition of PEG would also be advisible to selectively activate B-cells specific for the ctMPER helix sequence, and limit activation of B cells cross-reacting with the lipid bilayer.

However, the HVTN 133 study was stopped after detection of an anaphylaxis reaction in one participant, which was attributed to PEG. Our binding experiments suggest that the use of nonbilayer lipids such as PE and Chol should be avoided in PEG-free liposomal vaccines meant to initially activate ctMPER-specific responses. Nonselective insertion into lipid bilayers seems to be facilitated by the headgroup’s smaller cross-sectional area of these lipids.^35,52^ An alternative option would be the incorporation of SPM into the lipid composition. SPM imparts not only positive curvature to lipid bilayers, but also possesses both hydrogen bond donors and acceptors, its headgroup being capable of forming both inter- and intramolecular hydrogen bonds that contribute to increase lateral pressure at the membrane interface.^59^ Furthermore, at least in the case of the 10E8 Fab, SPM appears to occupy the PL-binding site identified in crystallography studies,^19^ indicating that this lipid may overall facilitate the epitope recognition process.

B cell lineages initially activated by ctMPER-antigens are expected to acquire higher affinity during maturation by selecting for changes that result in more favorable interactions with lipids.^24^ If recreating the native environment could be beneficial for that purpose, vaccines based on Chol-enriched thick-rigid VL membranes that contain ctMPER-TMD reconstituted could be the option of choice. However, the poor accessibility of the epitope in this system suggests otherwise. In conclusion, alternatives should be considered including the use of longer TMD scaffolds,^50^ or the substitution of Chol by nonbilayer lipids devoid of the capacity for enhancing bilayer stiffness.

## 4 Experimental section

### 4.1 Materials

Goat antimouse-AP antibody was purchased from Sigma (St. Louis, MO). The peptides ctMPER-TMD and ctMPER-TMD(Ala) spanning the gp41 MPER-TMD (Env residues 671–709, HXB2 numbering) (Figure S3) were synthesized as C-terminal carboxamides by solid-phase synthesis using Fmoc chemistry and purified by HPLC. Peptides were routinely dissolved in DMSO. Alkaline phosphatase conjugated anti-Fab and fluorescein isothiocyanate (FITC) labeled anti-Fab secondary antibodies were purchased from Sigma-Aldrich (St. Louis, MO). 1,1,1,3,3,3-hexafluoro-2-propanol (HFIP) was obtained from Sigma-Aldrich (St.Louis, MO, USA). The lipids 1-palmitoyl-2oleyl-*sn*-glycero-3-phosphocoline (POPC), 1-palmitoyl-2-oleoyl-*sn*-glycero-3-phosphoethanolamine (POPE), 1-palmitoyl-2-oleoyl-*sn*-glycero-3-phospho-l-serine (POPS), N-acyl-sphingosine-1-phosphorylcholine (SPM), cholesterol (Chol), 1-oleoyl-2-hydroxy-*sn*-glycero-3-phosphocholine (LPC), 1–2-dioleoyl-*sn*-glycerol (DAG) and 1,2-distearoyl-*sn*-glycero-3-phosphoethanolamine-N-[methoxy(polyethylene glycol)-2000] (PEG–PE) were purchased from Avanti Polar Lipids (Alabaster, Alabama). DPPE-StarRed was obtained from Abberior (Göttingen, Germany). DNA and protein concentrations were routinely determined at a nanodrop machine (Thermo scientific, Life technologies) by their absorbance at 260 and 280 nm, respectively. Dithiothreitol (DTT) was employed as a reducing agent for Fab labeling. The sulfhydryl-specific iodoacetamide derivative Fus4 was commercially available (Fisher Scientific) and Lin3 synthesized as previously described.^25^ Plasmids containing the genes for the expression of the Fabs were purchased from GenScript (New Jersey, U.S.A.) and Geneart (Thermo Fisher Scientific).

### 4.2 Computational Studies

A summary of the simulations conducted in this study is given in Table 1. The total simulation time of this study was approximately ten microseconds.

#### 4.2.1 System Set-ups & Molecular Dynamics Simulations

First, a complex model membrane bilayer consisting of POPC:POPE:PSM:POPS:CHOL, in a ratio 0.14:0.16:0.17:0.07:0.46, which mimics the viral membrane was constructed using the CHARMM-GUI membrane builder^32^ with a surface area of 100 × 100 Å^2^. The lipid bilayer was solvated to produce a simulation box with dimensions of 100 × 100 × 100 Å^3^, comprising approximately 94,000 atoms. The water–membrane system was minimized and equilibrated in multiple stages. During the first stage, the membrane was constrained, and water was allowed to be minimized for 5000 steps. During the second stage, the membrane was allowed to minimize for 5000 steps and the water was constrained. During the third stage, both water and membrane were minimized for 10,000 steps in the absence of constraints. Following minimization, 10 ns of NPT equilibration was carried out using the Nose-Hoover-Langevin piston to control the pressure with a 100 fs period, 50 fs damping constant and a desired value of 1 atm^60,61^ The system was coupled to a Langevin thermostat to sustain a temperature of 298 K. The software NAMD2.12 was employed to perform these molecular dynamics simulations.^62^

The preferred interactions and distributions of the phenyl-based linear compounds Lin3 and the polycyclic aromatic compound Fus4 were studied using the model membrane. Twenty-five small molecules were introduced randomly into the solution. In parallel, the X-ray crystal structure of the Fab-peptide complex (PDB ID 5GHW) at 2.40 Å resolution was employed in combination with the epitope of 10E8 that is noncovalently attached to Fab, and comprises a continuous helix spanning the gp41 MPER/transmembrane domain junction (MPER-N-TMD) including residues 671–687. Default protonation states were used for ionizable residues. The epitope was embedded in the complex viral-like bilayer. The Fab-peptide orientation observed in the crystal structure was preserved. The bilayer was built using the CHARMM-GUI membrane builder module as described earlier.^32^ The system was then solvated and neutralized using a 0.15 M KCl solution resulting in a simulation box of 170 × 170 × 160 Å^3^ dimensions. Two systems were built by mutating Ser65 of the antibody. Ser65 is a residue located away from the epitope binding pocket but it is suggested to be at the membrane interface and to insert partially into the membrane upon binding to the epitope. Following the experimental protocol, a chemical modification was first engineered to contain a single Cys residue at this position, and subsequently, two different classes of synthetic aromatic compounds for antibody modification were used: a phenyl moiety linked via a flexible spacer designated Lin3 and a polycyclic aromatic compound with a pyrenyl group, Fus4. Each of these systems comprised approximately 420,000 atoms.

The CHARMM36 force field was used to describe the Fab-peptide complex and lipids,^63^ the TIP3P model was used for water,^64^ and standard parameters were used for ions. The force field for the non-natural amino acids formed by the S-(2-amino-2-oxoethyl)-l-cysteine moiety with Lin3 or Fus4 attached were obtained by combining existing parameters and generating missing ones. Parameters for the free pyrene (Fus4) and 1,4-dyphenilbenzene (Lin3) had already been employed in^25^, and parameters for the S-(2-amino-2-oxoethyl)-l-cysteine moiety were already available in the additive CHARMM36 force field for nonstandard amino acids. The bond, angle and dihedral parameters with the exception of three, were generated automatically using the CHARMM-GUI Ligand Reader & Modeler module^65^ using the CHARMM general force field by chemical analogy and adopted unmodified. Three dihedral angles with high penalties: CG321-SG311-CG321-CG201, SG311-CG32-CG201-NG2S1, SG311-CG321-CG201-OG2D1 (in CHARMM force field notation) together with the charges were optimized following standard protocols using the FFTK VMD plugin.

The Particle Mesh Ewald method was used for the treatment of periodic electrostatic interactions, with an upper threshold of 1 Å for grid spacing.^66^ Electrostatic and van der Waals forces were calculated every time step. A cutoff distance of 12 Å was used for van der Waals forces. A switching distance of 10 Å was chosen to smoothly truncate the nonbonded interactions. Only atoms in a Verlet pair list with a cutoff distance of 16 Å (reassigned every 20 steps) were considered.^67^ The LINCS algorithm^68^ was used to constrain all bonds involving hydrogen atoms to allow the use of a 2 fs time step throughout the simulation. The multitime step algorithm Verlet-I/r-RESPA^69^ was used to integrate the equations of motion. The Nose-Hoover-Langevin piston method was employed to control the pressure with a 100 fs period, 50 fs damping constant and a desired value of 1 atm^60,61^ The system was coupled to a Langevin thermostat to sustain a temperature of 310 K throughout. The systems with the antibody were minimized using 10,000 steps and the steepest descent algorithm with an energy steep tolerance of 1,000 kJ mol^–1^nm^–1^. The equilibration consisted of six sequential steps in which the restraints of the protein backbone and side chain atoms, the lipid headgroups, and lipid torsions were progressively turned off. The first three steps of the equilibration were run for 125 ps at constant volume using -fs time step using sequential harmonic restraints on the protein backbone atoms of 4000, 2000, and 1000 kJ·mol^–1^·nm^–2^, followed by sequential harmonic restraints on the protein side chain atoms of 2000, 1000, and 500 kJ·mol^–1^·nm^–2^ at lipid headgroup atoms 1000, 400, and 400 kJ·mol^–1^·nm^–2^. Finally, restraints of 1000, 400, and 200 kJ·mol^–1^·rad^–2^ were used for the lipid torsions. Subsequently, simulations of 250 ps at constant pressure with a 2 fs time step were run with sequential harmonic restraint reduction at protein backbone atoms, protein side chain atoms, lipid headgroups, and torsions using force constants of 500 and 200 kJ·mol^–1^·nm^–2^, 200 and 50 kJ·mol^–1^·nm^–2^, 200 and 40 kJ·mol^–1^·nm^–2^ and 200 and 100 kJ·mol^–1^·rad^–2^respectively. Unconstrained dynamics was then performed for nearly one microsecond for each system in triplicates. Simulations were performed with Gromacs 2020.4^70^ and analysis was performed using in-house TCL scripts. The total simulation time was around 14 μs (Table 1).

#### 4.2.2 Metadynamics Simulations

NAMD 2.13 with plumed 2.13 were used to perform well-tempered metadynamics simulations to measure the potential of mean force of the small molecules crossing the viral-like model membrane. Each small molecule was placed in a different position along the x-plane of the membrane in each of the five replicas employed in the metadynamics run to account for the heterogeneity of the bilayer. The starting points were unbiased snapshots from MD simulations. For each of these replicas, five parallel walkers were employed with the small molecules starting at different positions along the *z*-axis equally separated. The distance between the center of the bilayer and the center of mass of the small molecule was the only biased collective variable. The center of the bilayer was defined using the average position of all the phosphorus atoms in the upper and lower leaflets. The Gaussian height and width were chosen to be 0.2 kcal mol^–1^ and 0.25 Å respectively with a deposition frequency of 500 steps. The bias factor, which regulates how fast the Gaussian height decreases, was set to 10 and the temperature to 298 K.

The position of the center of mass of the small molecule considered was constrained by a cylindrical potential perpendicular to the bilayer plane using an upper and a lower wall of 3 Å and centered considering the initial position of the small molecule in the replica. Some other collective variables were recorded and analyzed, such as rotation angles of the small molecules with respect to an axis perpendicular to the membrane plane.

### 4.3 Production and Site-Specific Chemical Modification of Abs

Experimental procedures described in^25^ were followed for the mutation, expression, purification and TCM of Fabs. Mutants bearing Cys residues at defined positions were subsequently modified with sulfhydryl-specific iodoacetamide derivatives of the aromatic compounds Fus-4 and Lin-3. Conjugation was monitored by matrix-assisted laser desorption and ionization (MALDI) mass spectrometry.

For the production of Fab-mVenus, sequences encoding for the heavy chain (HC) of the 10E8 Fab fused to mVenus and the Fab light chain (LC) were cloned in the pHLsec expression vector and produced in HEK293-F cells. A total of 20 μg of the LC plasmid was cotransfected with 40 μg of the HC into 200 mL of HEK293-F cells using PEIpro (Polyplus Transfections) at a 1:3 ratio of DNA: PEIpro. Cells were transfected at a cell density of 0.8 × 10^6^ cells/mL and incubated in an orbital shaker at 37 ◦C, 125 rpm and 8% CO2 for 7 days. The cells were harvested, and supernatants were retained and filtered with a 0.22 μm membrane (EMD Millipore). Supernatants were flowed through a protein A affinity column (GE Healthcare) by using an AKTA Start chromatography system (GE Healthcare). The column was washed with 20 mM Tris at pH 8.0,and 150 mM NaCl and eluted with 100 mM glycine at pH 2.2. Eluted fractions were immediately neutralized with 1 M Tris-HCl at pH 9.0. The fractions containing protein were mixed, concentrated, and flowed on a Superdex 200 Increase gel filtration column (GE Healthcare) to obtain purified samples and stored in a buffer containing 10 mM sodium phosphate (pH 7.5), 150 mM NaCl and 10% glycerol.

10E8 and 10E8-mVenus S65C Fabs were conjugated with either Fus4 or Lin3 molecules. For that, the Fab buffer was exchanged to the labeling buffer (10 mM NaH_2_PO_4_, 1 mM EDTA, pH 7.0), they were concentrated to at least 1 mg/mL, and 1 mM DTT was added to reduce free cysteine residues. After incubation at 37 °C for 30 min, Fus4 or Lin3 molecules were added in a 10x molar excess, and incubated overnight at 37 °C. After 16h, unconjugated compounds were removed using a PD-10 column to PBS + 10% glycerol.

### 4.4 Pseudovirus Production and Cell-Entry Assays

JRCSF (Clade B, tier 2) pseudoviruses (PsV) were produced by transfection of human kidney HEK293-T cells with three plasmids: (1) pWXLP-GFP, encoding a green fluorescent protein (GFP); (2) pCMV8.91, an Env-deficient HIV-1 genome; and (3) the full-length Env clone JRCSF (provided by Jamie K. Scott and Naveed Gulzar, Simon Fraser University, BC, Canada). The plasmids were used at a 2:1:1 ratio, respectively, in a total of 36 μg of DNA per plate. For the transfection, DNA was mixed with CaCl_2_ in HBS (HEPES buffer saline, pH 7.4), vortexed and incubated for 15 min at RT. The DNA-CaCl_2_ mixture was added to the cells, and incubated overnight at 37 °C. The following day, the media was changed to Opti-MEM and the plates were incubated at 33 °C for 48 h. Next, the media was retrieved and centrifuged at 500*g* for 5 min at 20 °C. The supernatant was filtered through 0.45 μm filters, transferred to ultracentrifugation tubes with a 20% sucrose in PBS cushion, and centrifuged at 100,000*g* in a swinging bucket rotor. The pellet was resuspended in 10 mM sodium phosphate (pH 7.5) and 150 mM NaCl with agitation for 30 min, and the recovered PsV were aliquoted and stored at −80 °C.

Cell-entry inhibition activity of Fabs was determined using CD4 + CXCR4 + CCR5 + TZM-bl target cells (ARRRP, contributed by J. Kappes). Cells were grown in DMEM High Glucose media +2 mM l-glutamine growth media, completed with 10% inactive FBS (Fetal Bovine Serum, inactivated at 56 °C) and 50 μg/mL gentamicin, and incubated at 37 °C, 5% CO2. Serial dilutions (1:3) of the Fabs were incubated with 10–12% infecting dose of PsV for 1, 5h min in 96 well plates. After incubation, 11.000 cells/well of TZM-bl cells were seeded in the well (supplemented with 25 μg/mL dextrans (Sigma-Aldrich, Steinheim, Germany)). After 72h, the number of infected cells expressing GFP was determined by Flow Cytometry (Cytoflex S, Beckman Coulter, IN, USA). IC_50_ values (the Fab concentration needed for a 50% inhibition of the infection) were calculated by performing a nonlinear fitting of the experimental inhibition vs Fab concentration values using GraphPad Prism.

### 4.5 GUV Production and Binding Assay

Giant Unilamellar vesicles (GUVs) were produced following the electro-formation method. A total of 2 mM of lipid was dissolved in CHCl_3_, in a final volume of 100 μL with the fluorescent probe DPPE-Star Red (2%). When required, the ctMPER-TMD or ctMPER-TMD (Ala) peptide dissolved in 10% (v/v) HFIP was included in the organic phase at 1:250 peptide-to-lipid ratio. The following GUV compositions were used: POPC ± PEG, POPC:Chol at a 2:1 ratio, POPC:POPE at a 2:1 ratio, POPC:POPS at a 2:1 ratio, POPC:SPM at a 2:1 ratio ± PEG, POPC:DAG at a 9:1 ratio, and POPC:LPC at a ratio 9:1, Viral-like composition (VL) ± PEG (14 POPC%, 16 POPE%, 7 POPS%, 17 SPM%, and 46% Chol) and plasma membrane-like (PML) composition ± PEG (POPC:Chol:SPM at a ratio 2:2:1). Four μL of the lipid stock were added to platinum electrodes, and the wires were introduced into a specially designed chamber (Industrias Técnicas ITC, Bilbao, Spain), containing 400 μL of a 300 mM sucrose solution, previously equilibrated to RT. The lipid mixtures were incubated for 1h and 45 min in a waveform generator (Siglent SDG1032X) at 10 Hz, resulting in the generation of vesicles containing sucrose, and 1h at 2 Hz, to initiate the detachment of the GUVs from the electrodes. The GUVs were subsequently transferred to a BSA-blocked microscope chamber and incubated for 15 min with 250 nM 10E8 Fab-mVenus WT, or the conjugated versions.

The images were acquired on a Leica TCS SP5 II microscope (Leica Microsystems GmbH, Wetzlar, Germany). GUVs were excited at 633 nm by using a HeNe laser, and emission was imaged at 665 ± 30 nm by using a 63× water immersion objective (numerical aperture (NA) = 1.2). Relative intensity values of Fab–GUV binding were obtained by measuring the fluorescence intensity of mVenus (excited at 476 nm, and emission was imaged at 535 ± 15 nm) along the equatorial plane of the GUV images, in a number of vesicles n ≥ 20. Images were processed with Fiji ImageJ software, to achieve relative fluorescence intensity values (Figure 2C), and results were plotted and statistically analyzed with GraphPad Prism software, using a two-way ANOVA test.

### 4.6 Flow Cytometry

Stable HEK293T cell lines expressing HIV Env Comb-mut (ADA.CM.V4) and the MPER-TM_654–709_ polypeptide were previously described.^41,42^ A total of 10^6^ cells were washed in FACS buffer. (FACS buffer is PBS supplemented with 0.1% heat-inactivated fetal bovine serum and was used throughout the experiment). Fab-mVenus variants were added at a concentration of 2 μg/mL for 30 min in the dark at RT while rocking. In some cases, Ig-mD1.22 was added to cells at a concentration of 20 μg/mL and the treated cells were incubated for 30 min prior to incubating cells with the antibodies. Ig-mD1.22 is an immunoadhesin that was designed and produced in-house and consists of a modified single domain of human CD4, mD1.22,^43^ which exhibits high expression and thermal stability, fused to a human IgG1 Fc domain. Cells were spun down at 1000 rcf for 1 min, washed twice, and then resuspended in FACS buffer for analysis. During the last 15 min of incubation, cells were stained with Fixable Aqua Live Dead Cell Stain (Life Technologies L34957). Cells were acquired and analyzed using NovoCyte (ACEA Biosciences Inc.). Data were analyzed using FlowJo v10.8 (BD Life Sciences).

## Supporting Information Available

It includes effects of nonbilayer lipids DAG and LPC on Fab binding, Flow cytometry gating strategy followed, ctMPER-TMD sequence designation, binding of WT Fab to ctMPER-TMD helix reconstituted in membranes, lipid contacts established by free forms of Fus4 and Lin3 and main monolayer curvatures after Fab insertion (PDF)
