# Supplementary material for: Generation of a Nonbilayer Lipid Nanoenvironment after Epitope Binding Potentiates Neutralizing HIV-1 MPER Antibody
Source: ACS Appl Mater Interfaces. 2024 Oct 24;16(44):59934–48. doi: 10.1021/acsami.4c13353 (PMC11551957; doi:10.1021/acsami.4c13353)
Supplement: Supplementary file 1 — am4c13353_si_001.pdf [file am4c13353_si_001.pdf]

Supporting Information

Generation of a nonbilayer lipid nanoenvironment after epitope binding potentiates neutralizing HIV-1 MPER antibody

Sara Insausti[1,2#], Ander Ramos-Caballero[1#], Brian Wiley[3], Saul González-Resines[3], Johana Torralba[1,2], Anne Elizaga-Lara[1,2], Christine Shamblin[4], Akio Ojida[5], Jose M. M. Caaveiro[6], Michael B. Zwick[4], Edurne Rujas[1,7,8], Carmen Domene[3*], and José L. Nieva[1,2*]

[1]Instituto Biofisika (CSIC, UPV/EHU), University of the Basque Country (UPV/EHU), P.O. Box 644, 48080 Bilbao, Spain

[2]Department of Biochemistry and Molecular Biology, University of the Basque Country (UPV/EHU), P.O. Box 644, 48080 Bilbao, Spain

[3]Department of Chemistry, University of Bath, Claverton Down, Bath, BA2 7AX, United Kingdom.

[4]Department of Immunology and Microbiology, The Scripps Research Institute, La Jolla, California, CA 92037, USA

[5]Department of Chemical Biology, School of Pharmaceutical Sciences, Kyushu University, Fukuoka 819-0395, Japan

[6]Laboratory of Protein Drug Discovery, School of Pharmaceutical Sciences, Kyushu University, Fukuoka 819-0395, Japan

[7]Department of Pharmacy and Food Sciences, Faculty of Pharmacy, University of the Basque Country (UPV/EHU), 01006 Vitoria, Spain

[8]Ikerbasque, Basque Foundation for Science, 48013 Bilbao, Spain

[#]These authors contributed equally
*Correspondence: JLN (email: joseluis.nieva@ehu.eus) and CD (email: C.Domene@bath.ac.uk)

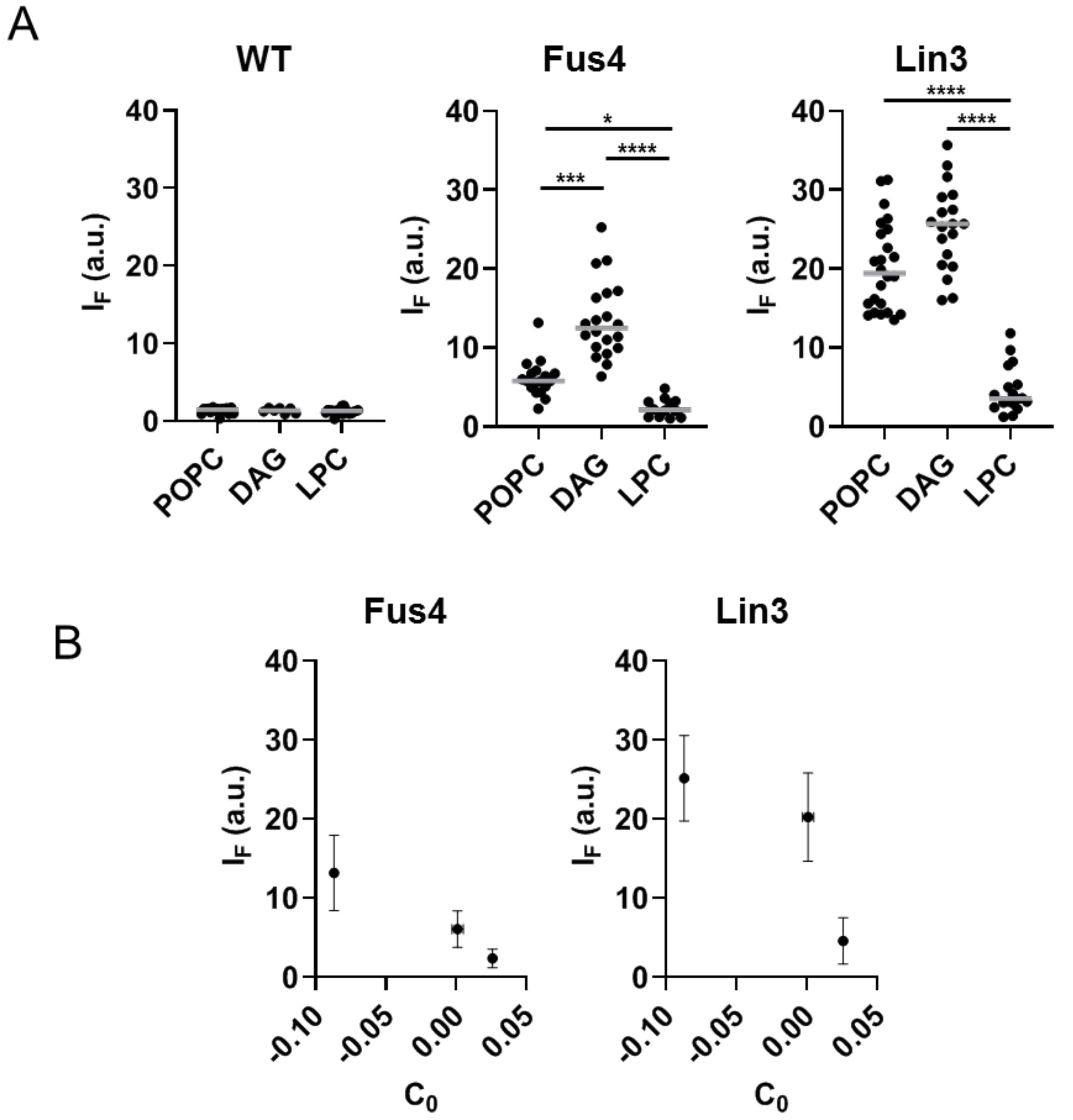

Figure S1: Effect of nonbilayer lipids on binding of Fab 10E8 and chemically modified versions to POPC bilayers. A) Binding to single vesicles. The nonbilayer lipids DAG or LPC were added to a 10 mole % concentration. B) Dependence of binding on the spontaneous curvature of added lipids. Conditions otherwise as described in Figure 2 of the main text. (****p<0.0001, ***p<0.0002, *p<0.03).

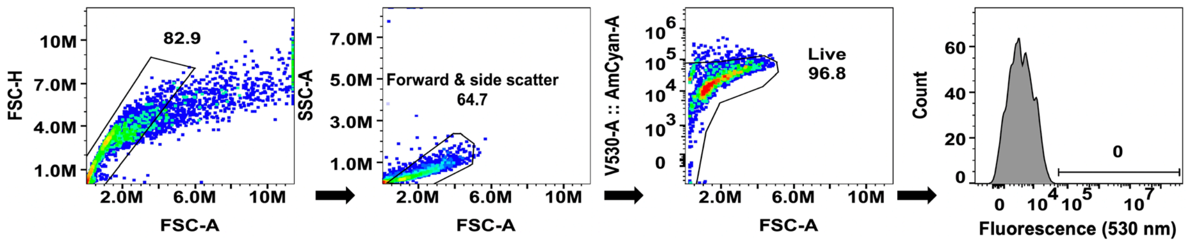

Figure S2: Flow cytometry gating strategy. HEK293T cells (and related stable cell lines bearing MPER antigens) were gated as shown to remove doublets and to discriminate cellular populations from debris. Live cell populations were determined and the final gate was positioned on a histogram of the relative fluorescence acquired using HEK293T cells as the negative control cells.

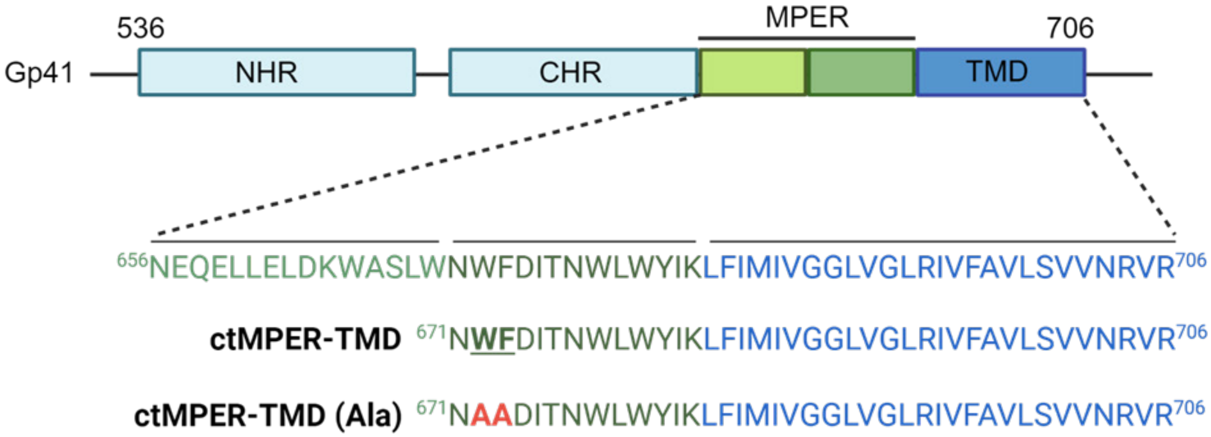

Figure S3: Designation of the ctMPER-TMD sequences used in this study to test binding specificity to ctMPER reconstituted in membranes.

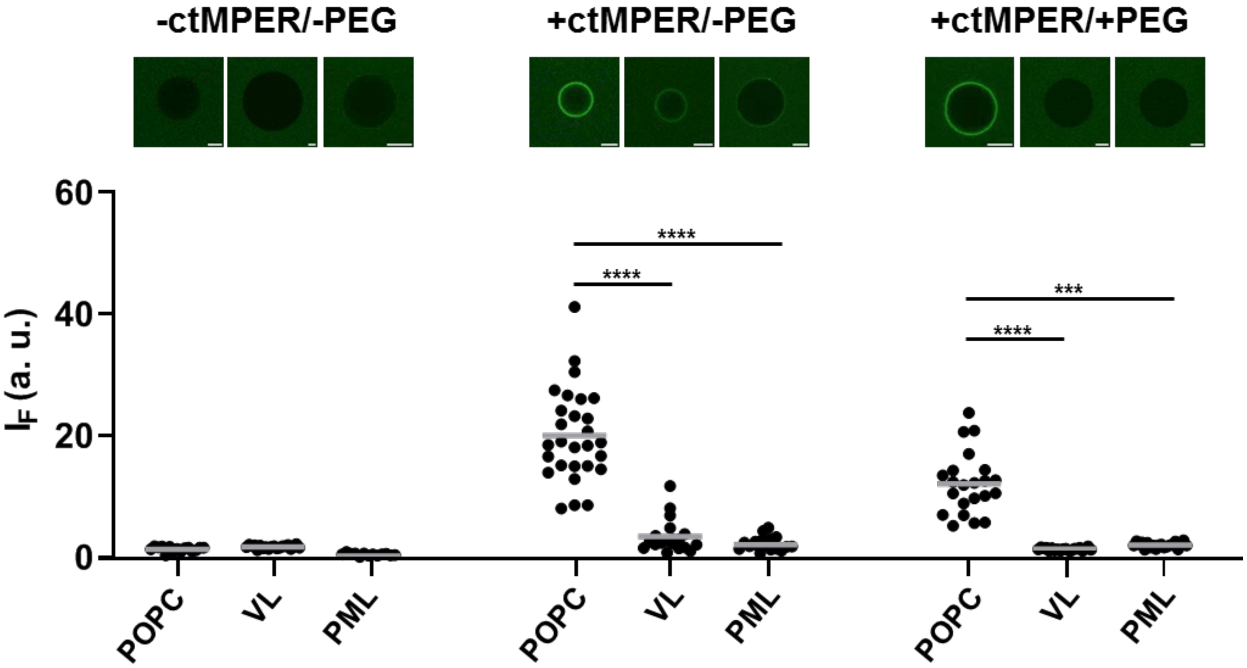

Figure S4: Recognition of ctMPER-TMD helix reconstituted in membranes by WT Fab 10E8. The plot compares binding of the Fab-mVenus chimera to POPC, PML or VL vesicles. Substantial binding is only detected in POPC vesicles that contained the ctMPER-TMD helix, even in the presence of PEG-PE (5%). (*****p*<0.0001, ****p*<0.0002)

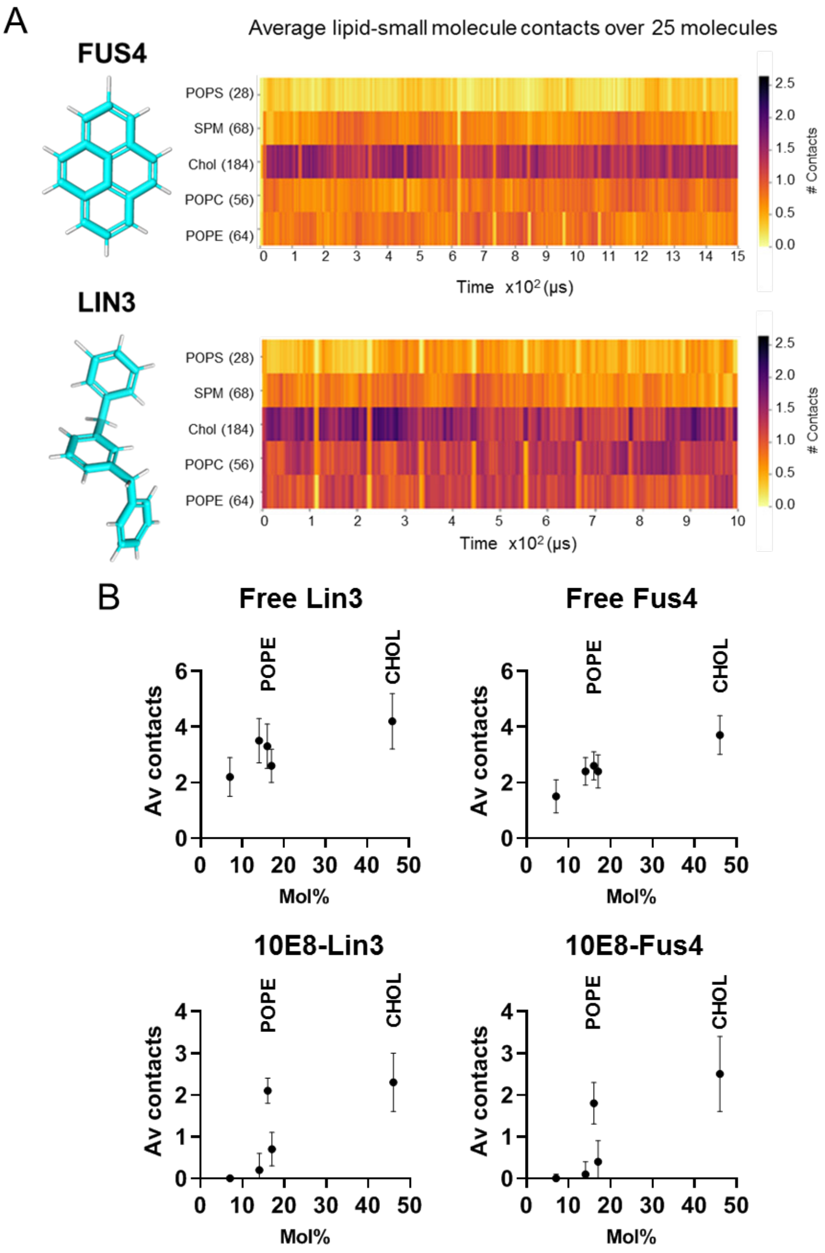

Figure S5: Lipid contacts established by free forms of Fus4 and Lin3 in VL membranes. A) Average contact maps between 25 small molecules and the components of the model viral membrane from microsecond atomistic MD simulation trajectories. A contact is considered when the distance between any heavy atom of the small molecule from any heavy atom of a lipid residue is ≤3.5 Å. The number of specific lipids in the model membrane used in the simulation is indicated in brackets for each component along the y-axis. B) Plots displaying the dependence of the number of contacts on the mole ratio. For comparison the bottom panels display contacts by the compounds derivatized with Fab.

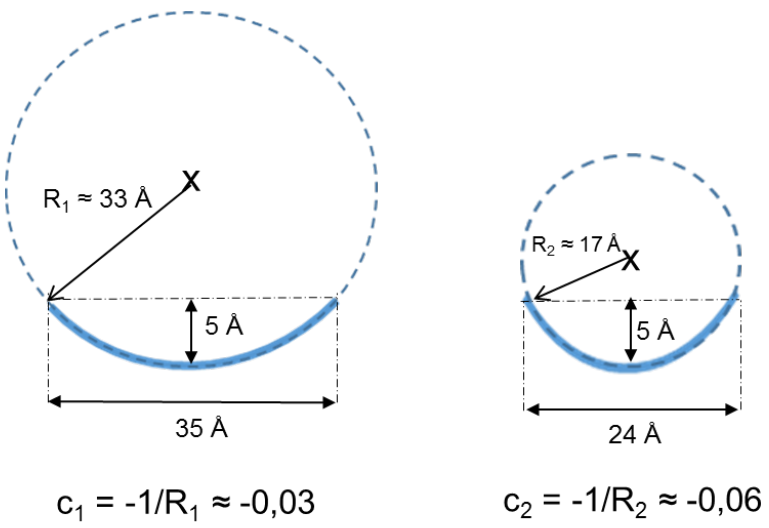

Figure S6: Main curvatures in the deformation generated by Fab insertion into the monolayer
